# Modelling the seismic potential of the Indo-Burman megathrust

**DOI:** 10.1038/s41598-021-00586-y

**Published:** 2021-10-27

**Authors:** Inessa Vorobieva, Alexander Gorshkov, Prantik Mandal

**Affiliations:** 1 Institute of Earthquake Prediction Theory and Mathematical Geophysics, Russian Academy of Sciences, 84/32 Profsouznaya, Moscow, Russia 117997; 2 CSIR-National Geophysical Research Institute, Uppal Road, Hyderabad, Telangana 500007 India

**Keywords:** Solid Earth sciences, Seismology

## Abstract

The Indo-Burman arc is the boundary between the India and Burma plates, north of the Sumatra–Andaman subduction zone. The existence of active subduction in the Indo-Burman arc is a debatable issue because the Indian plate converges very obliquely beneath the Burma plate. Recent GPS measurements in Bangladesh, Myanmar, and northeast India indicate 13–17 mm/y of plate convergence along a shallow dipping megathrust while most of the strike-slip motion occurs on several steep faults, consistent with patterns of strain partitioning at subduction zones. A short period of instrumentally recorded seismicity and sparse historical records are insufficient to assess the possibility of great earthquakes at the Indo-Burman megathrust. Using the advantage of the Block-and-Fault Dynamics model allowing simultaneous simulation of slow tectonic motions and earthquakes, we test the hypothesis whether the India-Burma detachment is locked and able to produce great earthquakes, or it slips aseismically? We have shown that the model of locked detachment is preferred because it more adequately reproduces observed tectonic velocities. The integral characteristics of synthetic seismicity, the earthquake size distribution, and the rate of seismic activity are consistent with those derived from observations. Our results suggest that the megathrust is locked and can generate great M8+ earthquakes. The estimated average return period of great events exceeds one thousand years. Earthquakes of this size pose a great threat to NE India, Bangladesh and Myanmar, the most densely populated areas of the world.

## Introduction

**Figure 1:**
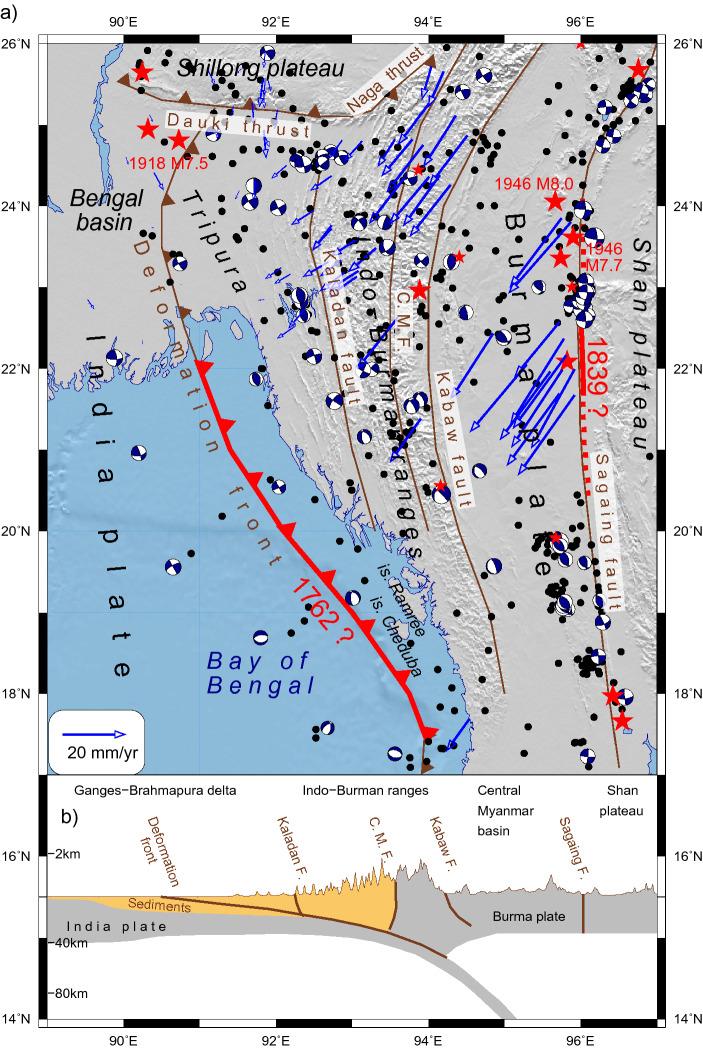
Comprehensive overview of the Indo-Burman megathrust. (**a**) Major faults^4^ are shown in brown, CMF is Churachandpur-Mao fault; Black dots and red stars mark shallow (h < 40 km) earthquakes with M ≥ 4.5 (1973–2020) and M ≥ 6.5 (1900–2007) (ANSS, Centennial catalog^9^). Beach-balls are Fault Plane Solutions (FPS) (GCMT); blue arrows show GPS velocities^3,4,17,32–34^ relatively to stable India (Table S1). Large earthquakes discussed in text are labeled. Possible ruptures of the 1762 Arakan earthquake^12^ and the 1839 Ava earthquake^8^ are traced by red. (**b**) Schematic cross-section at latitude 24°N, modified after^4^.

The Indo-Burman region is a prominent earthquake-prone area, where seismicity is governed by subduction of the Indian Plate beneath the Burma plate at a gentle angle (Fig. 1). This convergence is accommodated by the Indo-Burman megathrust, which resulted from an oblique collision in the Miocene^1^. According to geologic and seismic data, the megathrust is uncommonly broad and shallow^2^. On the surface, the megathrust is expressed by the folded Tripura lowland and the highly deformed Indo-Burman Ranges (IBR). The subduction is going along the gently dipping Indo-Burman Detachment (IBD). Recent observations from the Indian, Myanmar and Bangladesh GPS networks^3,4^ suggest that the shortening rate across the Burma plate is 12–24 mm/y and 13–17 mm/y, respectively. Based on the relative motion of India and the undeformed Sundaland block, Panda et al.^5^ found that convergence across the blind megathrust (~ 7 mm/y) is significantly lower than previously thought^3,4^. They exclude the influence of the toroidal flow from Eastern Tibet, which induces additional arc-normal motion of ~ 10 mm/y in the non-rigid Shan Plateau. The estimates of total dextral shear rate vary from 25 to 42 mm/y^3,4–7^, it occurs along the Sagaing fault at the eastern margin of the Myanmar Central Basin and along the Churachandpur-Mao fault (CMF), and other faults.

The shallow earthquakes occur in response to the partitioning of the India-Eurasia motion along two distinct boundaries viz., the accretionary wedge and IBR in the west, and the Sagaing Fault in the east (Fig. 1). Most of the large earthquakes are associated with the right-lateral strike-slip Sagaing Fault that has produced several large instrumental and historical events. The 1839 Ava earthquake was a catastrophic disaster that struck Burma on March 23 (Fig. 1a). Its macroseismic intensity was estimated in the range from IX to XI^8,9^. Historical records indicated that the earthquake devastated the ancient capital of Ava with a rupture length as long as 400 km^7^. The pair of the 1946 Sagaing earthquakes (i.e. the Wuntho earthquakes) occurred on September 12 (Fig. 1a). The mainshock *M*_w_8 (ANSS) was followed three minutes later by a second one *M*_w_7.7 south of the mainshock. Different agencies reported estimates of the mainshock magnitude ranging from *M*_w_8 (ANSS) to *M*_w_7.3^9,10^. Taken together, the Sagaing earthquakes could have ruptured a segment of the fault over 300 km in length^9,11^. Additionally, the Sagaing Fault was ruptured by several shocks with *M* ≥ 7.

The present-day seismicity in Tripura is not significant and occurs mainly on steep shallow strike-slip faults, but the focal mechanisms of some earthquakes are consistent with a rupture along the gently dipping IBD^12,13^. However, historical records report the great 1762 Arakan earthquake, which triggered a local tsunami in the Bay of Bengal^14^ and was associated with extended areas of uplift and subsidence^15^. The Arakan earthquake had the extreme macroseismic intensity of XI. Its likely location is varying from near Chittagong to along the Arakan coast. The extent of the rupture might have been as much as 700 km along with the plate interface. The 700 km extent combined with a displacement of 10 m gives a maximum estimated magnitude of *M*_w_8.8^16^.

Meanwhile, the possibility of great earthquakes in the northern segment of the Indo-Burman megathrust is still a matter of some controversy due to the relatively low level of present-day seismicity^17,18^. The principal issues to be addressed here are as follows:

(i) Whether the observed shortening across the Burma plate is being released by aseismic slip on the IBD?
(ii) Whether the megathrust is locked and presently accumulating strain that will be probably released by future earthquakes?

The goal of the present work is to evaluate the possibility of great earthquakes in the Indo-Burman megathrust. For this purpose, we exploit the Block-and-Fault Dynamics Model (BAFD) that was developed to simulate regional lithosphere dynamics and seismicity^19–21^. The model was designed under the hypothesis that the structure of a region, fault kinematics, and the statistics of regional seismicity are fundamentally interrelated. The region is modelled as a system of rigid crustal blocks separated by thin visco-elastic faults, which move in response to external tectonic and basal motions. Several static block models were recently developed^3–5^ to study the capability of the Indo-Burman megathrust of generating great earthquakes. The basic principles of elastic dislocation models^3–5^ differ significantly from those of the BAFD model. The advantage of our dynamic model is that it simulates both slow tectonic motions and earthquake sequences. It allows studying a wide range of problems from testing of geodynamic hypotheses to seismic risk assessment^21–26^.

We have outlined the block structure of the Indo-Burman megathrust based on the tectonic structure and major regional faults. External motions are specified according to the available GPS measurements in the areas around the block structure. Through numerical experiments, we simulate the regional dynamics and seismicity under the assumption that the Indo-Burman megathrust is locked or is sliding aseismically. We are looking for the preferred model that most adequately reproduces the observed tectonic motions and seismicity patterns inside the block structure.

## Tectonic settings and the block-structure of the Indo-Burman megathrust and numerical parameters of modelling

The N–S trending Indo-Burman megathrust runs along the subduction/convergence boundary between the Indian and Burma plates (Fig. 1). It joins the eastern Himalaya syntax in the north and the Andaman-Sumatra subduction zone in the south. Major subduction events took place in the region from the late Lower Cretaceous to Mid-Miocene and Quaternary periods, finally building the complex tectonic structure^27^. From west to east, the major tectonic structures are (1) the Tripura wedge composed of Neogene folded sedimentary rocks, (2) a system of the arched Indo-Burman Ranges (IBR) composed of strongly deformed Tertiary rocks, (3) the Paleogene—Neogene sedimentary Central Myanmar Basin showing Tertiary and Quaternary volcanism, and (4) the highly elevated Shan Plateau consisting of crystalline rocks. Their origin and evolution were determined by the compressional stresses caused by the highly oblique convergence of the India plate subducting beneath the Burma plate^28,29^.

The megathrust considered in our modelling encompasses the region laying between the Bengal basin in the west and the Central Myanmar Basins in the east. The zone of the megathrust includes the Tripura folded area of low topography and the elevated IBR. Numerous arcuate faults dominate the inner fold-and-thrust structure of the IBR^8,30^.

We outline the block structure along deformation zones marked by the mapped regional faults that have complex structures^8,30,31^. The Deformation Front is the western boundary of the modelled block structure (Fig. 1). The Kabaw fault zone limits the block structure to the east. The Dauki and Naga thrusts form the northern boundary. In the south, the simplified EW boundary separates the modelled area from the Andaman subduction zone. While the southern boundary does not correspond to a mapped fault, it does correspond to southern end of 1762 rupture and where the plate boundary bends.

Three blocks delineated within the megathrust zone compose the block structure for the BAFD modelling. The block boundaries, the geological structures and topography are uniform within each block. The Tripura block includes the area of low topography between the Deformation front and the Kaladan fault zone. The second block encompasses the western Indo-Burman Ranges and is bounded by the Kaladan fault zone to the west and by the CMF zone to the east. The third block includes the eastern Indo-Burman Ranges and is limited by the CMF and Kabaw zones (Figure S1). Notable transverse structures are not reported across Indo-Burman arc and segmentation simply reproduces the arcuate shape of fault zones.

We did not include in the block structure the Sagaing fault whose high seismic potential has been attested by the occurrences of several large instrumental and historical events (Fig. 1). We focus on the western margin of the Burma plate, the seismic potential of which is still unknown.

The style of faulting is not an input parameter of the BAFD model, but is the result of simulation. Dip angles of faults may influence the focal mechanism of modeled earthquakes and distribution of strain among fault zones. Usually, gentle faults accommodate shortening, while steep faults release strike-slip deformation. Dip angles of faults are prescribed based on the cross-sections^4,12^. The CMF is a sub-vertical fault with a dip angle of 80°. The Kabaw and Kaladan faults having the complex structure reveal both strike-slip and thrust features^8,30^. We model them as steeply inclined faults with a dip angle of 60°.The dip angle of the IBD changes from 10° in the north to 15° in the south. The depth of blocks is assumed to be 30 km which corresponds to the locked depth of the IBD in models^3,4^. The extension of the locked portion of IBD roughly coincides with the location of the Kaladan fault^3,4^. (Table S3).

External velocities at the lateral boundaries are specified based on the GPS observations ^3,4,17,32–34^ in the territories surrounding the block structure (Tables S1, S2, Figure S4). The velocity of the India plate is *V*_*E*_ = 0; *V*_*N*_ = 0 mm/y while the velocity of the Burma plate is *V*_*E*_ = −17; *V*_*N*_ = −22 mm/y. The basal velocities are unknown since direct measurement is not possible at depth. Our choice is based on the cross-section ^4^ and GPS observations on the surface. Arakan-Tripura and western Indo-Burman ranges (Blocks 1 and 2) overlap the Indian plate, so the basal velocity is the same as the velocity of India, namely *V*_*E*_ = 0; *V*_*N*_ = 0 mm/y. Eastern Indo-Burman ranges (Block 3) overlap the Burma plate, and the basal velocity is the same as the GPS velocity of the Burma plate measured in the Central Myanmar Basin, *V*_*E*_ = −17; *V*_*N*_ = −22 mm/y.

The rheology and parameters that control the occurrence of earthquakes are poorly constrained by observations and models, e.g.^35,36^. We prescribe the values based on our experience in the BAFD modelling. The elastic parameter of the faults and the bottom of the blocks is 1 bar/cm. The factor for the rate of inelastic displacements is $$2 \times 10^{ - 4} {\raise0.7ex\hbox{${{\text{cm}}}$} \!\mathord{\left/ {\vphantom {{{\text{cm}}} {{\text{bar}} \cdot \,{\text{y}}}}}\right.\kern-\nulldelimiterspace} \!\lower0.7ex\hbox{${{\text{bar}} \cdot \,{\text{y}}}$}}$$ in locked faults, and $$2 \times 10^{ - 3} {\raise0.7ex\hbox{${{\text{cm}}}$} \!\mathord{\left/ {\vphantom {{{\text{cm}}} {{\text{bar}} \cdot \,{\text{y}}}}}\right.\kern-\nulldelimiterspace} \!\lower0.7ex\hbox{${{\text{bar}} \cdot \,{\text{y}}}$}}$$ in slipping faults as well as block bottoms. The friction coefficient is considered to be 0.5, and the difference between lithostatic and hydrostatic pressure is assumed to be 3 Kbar. We model for a time period of 20 thousand years with a time step of 0.04 y. The size of the cell discretizing fault segments is 2 km that allows the seismicity modelling from the lowest magnitude of ~ 4.7. Table S4 contains the full list of the parameters. Locked/unlocked faults are modelled by changing the inelastic rate factor. All other parameters are fixed.

## Results

### Selection of the preferred model

We performed three numerical experiments (EX1–3) to study the dynamics of the Indo-Burman arc depending on the style of stress release in the megathrust:

- EX1: the IBD is locked along with the entire extent;
- EX2: the IBD is unlocked;
- EX3: southern oceanic part of the IBD is locked, while the northern onshore part is unlocked.

All other faults composing the block structure are treated to be locked in the EX1–EX3. Finally, the preferred model from the above three cases was chosen based on the minimum residual between measured (Table S1) and simulated tectonic velocities of the inner blocks of the structure (Table S5).

**Figure 2:**
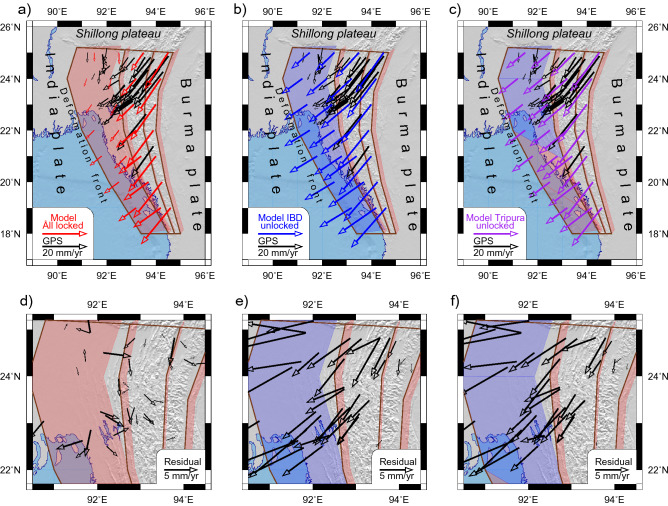
Block velocities modeled in three experiments. (**a**) Experiment 1 “Locked IBD” (red arrows); (**b**) Experiment 2 “Unlocked IBD” (blue arrows); (**c**) Experiment 3 “Partially locked IBD” (purple arrows). GPS velocities are shown by black arrows; locked and unlocked faults are highlighted by transparent red and blue. (**d**), (**e**) and (**f**) display the residual velocities for (**a**), (**b**) and (**c**), respectively.

The modelled and observed block motions show a good correlation when the IBD is locked (Fig. 2a,d). The RMS value, in this case, is 2.5 mm/yr. The velocity field is similar to that modeled in the study^3^, including clockwise rotation of blocks. The simulated velocities in the Arakan-Tripura and eastern IBR blocks are much larger than observed ones if IBD is completely or partially unlocked (Fig. 2b,c,e,f). The RMS values are 7.1 and 6.5 mm/y, respectively. Therefore, the model of the locked megathrust along its entire extent is preferred. Table S5 contains the full list of modelled block velocities and RMS.

**Figure 3:**
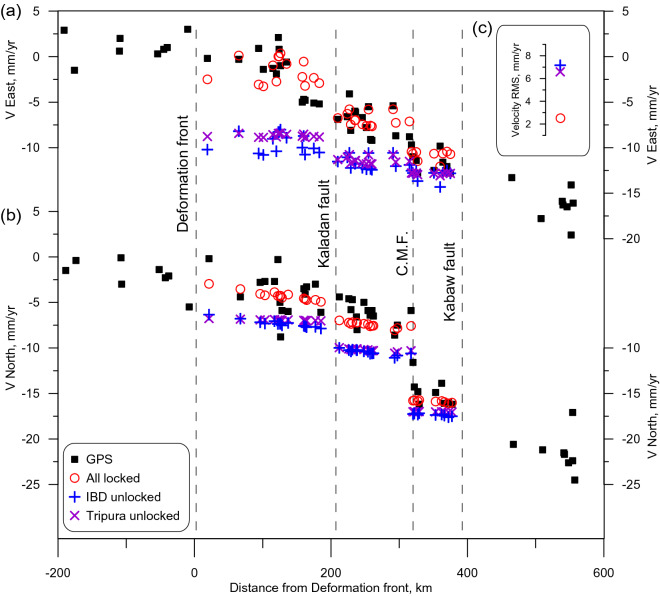
Changes of the modeled interseismic velocities across the Indo-Burman arc as simulated in three experiments. The values are given at the GPS sites, their locations in the plot are distances to the nearest fault. Fault locations (dashed lines) are as at 24°N. The modeled velocities are shown by colored symbols; observed GPS velocities (Table S1) are shown by black squares. (**a**) Eastward velocities; (**b**) Northward velocities; (**c**) Residual Mean Squared velocity obtained in the three experiments, symbols are the same as in (**a**) and (**b**).

Changes of the modelled interseismic velocities across the Indo-Burman arc are shown in Fig. 3 that illustrates the distribution of the EW shortening and NS shear deformation over the fault zones. It is only the preferred model, which reproduces GPS observations and strain portioning in the region. Most of the shear deformations concentrate in the CMF where there is a significant discontinuity in the observed and modelled velocities. Meanwhile, the shortening changes gradually across the arc, which points to accumulating slip deficit. The distribution of the shortening and shear deformation across the region are supported by similar results of study^4^. The observed and modelled eastward velocities in the Tripura are small. We will show later on that the shortening occurs here by seismic moment release in the IBD. This result is similar to one modelled by Panda et al.^5^.

We also studied the influence of coupling in the Kaladan, CMF, and Kabaw faults on the regional dynamics (EX4–6; Figures S5-S7, Table S5). Although the aseismic sliding on these faults has less impact on block movements compared with the IBD, the resulting RMS of the order of 3.1 to 5.1 mm/y is greater than in EX1. The velocities obtained with unlocked CMF (RMS 3.1 mm/y) differ little from the velocities in the preferred experiment and are quite consistent with the observations. Thus, the CMF properties have little impact on the regional dynamics. The fault slips modelled in the EX1–EX6 show shortening in the IBD and Kaladan, dextral shear slip in the CMF, and oblique convergence in the Kabaw. For each fault, the maximum slip rate occurs in the simulations when the fault is unlocked. (Figure S8).

### Synthetic seismicity

**Figure 4:**
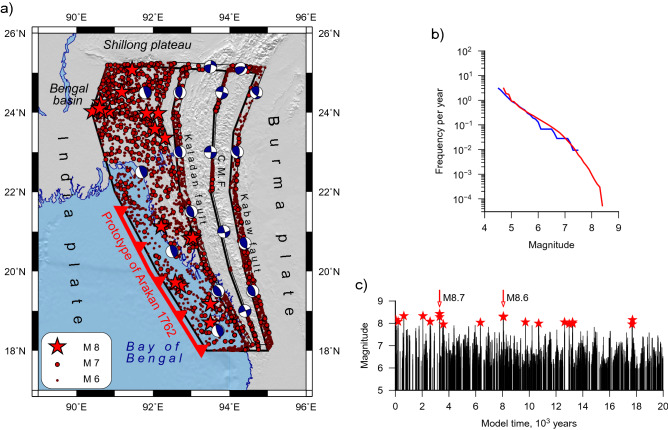
Overview of synthetic seismicity simulated for 20 thousand years by the preferred model “All faults locked”. (**a**) The map of epicenters: all great earthquakes with M ≥ 8 (red stars) are simulated in the India-Burma Detachment (IBD); beach balls are synthetic FPS. (**b**) Earthquake size distribution (frequency per year) for the synthetic (red) and recorded (blue) seismicity. (**c**) Time sequence of synthetic M6+ earthquakes. The rupture zone of two multi-segment giant M8.7 and *M*8.6 earthquakes is marked by red thrust line in (**a**), and by arrows in (**c**).

We analyzed the synthetic seismicity for a time period of 20 thousand years as generated by the preferred model (Fig. 4a–c). Among 65 thousand synthetic earthquakes, 19 events have a magnitude M ≥ 8. Most of the modelled epicenters (45 thousand) are concentrated on the IBD. All great synthetic events (M8+) occurred in the same area (Fig. 4a). The maximum magnitude of 8.4 is modelled in the Arakan section of IBD. The synthetic seismicity generated along the IBD shows an irregular pattern and periods of seismic activity are replaced by quiet periods when no earthquakes occur (Fig. 4c, S7). Such periods last up to several hundred years and may be associated with the present-day low seismic activity in the Arakan-Tripura area.

A large number of synthetic earthquakes occur on the Kaladan and Kabaw faults, while the level of seismic activity is relatively low on the CMF (Fig. 4a). The analysis of the portion of relative motion released by earthquakes shows high coupling on the IBD, Kaladan, and Kabaw faults, while in the central segment of CMF the coupling is low (Table S6, Figure S9). This result is consistent with Gahalaut et al.^17^.

The average recurrence time of M8+ earthquakes is about one thousand years, but great earthquakes do not come at regular intervals (Fig. 4c). The number of major synthetic shocks varies from 0 to 4 per millennium. The inter-event time can reach several thousand years (Fig. 4c). Two great earthquakes with *M* > 8 were modelled simultaneously on neighboring segments in the Arakan section of the IBD (Fig. 4a). They may be viewed as multi-segment mega-earthquakes with *M* = 8.7 and *M* = 8.6. These synthetic events may be treated as prototypes of the 1762 Arakan earthquake (Fig. 4a). Several M8+ shocks were simulated in the Tripura section where no events of this size are reported by the instrumental earthquake catalogues and historical records.

The earthquake rate and the size distribution (frequency per year) of the modeled seismicity are consistent with these features of the recorded seismicity (Fig. 4b). The plot for recorded shallow earthquakes (h ≤ 40 km) was constructed using events that occurred within 50 km of the block structure from ANSS (1973–2020), and for M6.5+ we included data from the Centennial catalog^9^ for the period 1900–1972. The typical synthetic source mechanisms show reverse faulting in the IBD, Kabaw and Kaladan faults while dextral strike-slip is modelled in the CMF and the northern segment of the Kabaw fault.

**Figure 5:**
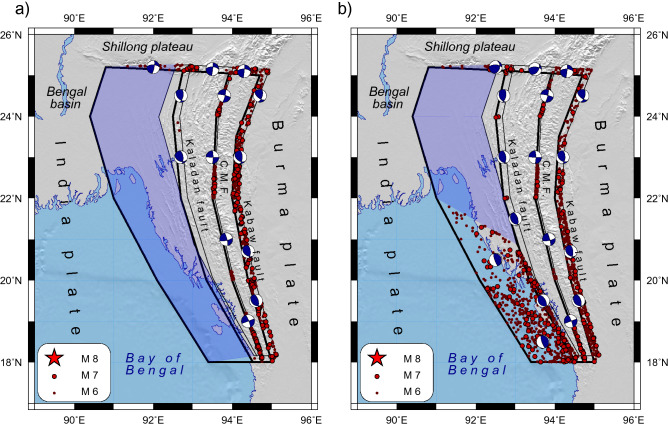
Map of synthetic seismicity simulated for 20 thousand years: (**a**) EX2 “IBD unlocked”; (**b**) EX3 “Tripura unlocked”. Unlocked segments are highlighted by transparent blue; other notations are like in Fig. 4.

The synthetic seismicity modelled with an unlocked IBD (EX2, 3, Figs. 5, S9, S10) shows a worse fit to the observations. The rate of the synthetic seismicity is 3–5 times lower than the observed one, and the spatial distributions of synthetic and observed epicenters are discrepant. The synthetic catalogs do not contain great M8+ shocks similar to the 1762 Arakan earthquake that contradict the historical records and paleoseismic studies^15,37^.

Figures S14-S16 display the changes in the seismicity patterns due to the unlocked Kaladan, CMF and Kabaw faults (EX4–6). The properties of the CMF (EX5, Figure S15) have little impact on the synthetic seismicity compared with the preferred experiment. With the unlocked CMF, the model generated sixteen earthquakes with M8+ in the IBD and two multi-segment mega-earthquakes like the 1762 Arakan event. Additionally, we obtained 4 mega-earthquakes in the onshore Tripura section of the IBD with maximum equivalent magnitudes ranging from 8.5 to 8.7. Maximum magnitudes modelled in all experiments are summarized in Table S7.

We performed an additional experiment for the block structure including the Sagaing fault and supposing all faults to be locked. The Central Myanmar basin is being transformed from an outer block to an inner one. By our expectation, this transformation has little effect on the interseismic block motions and the seismicity in the IBD and other faults. It is not surprising, because the motion of the Central Myanmar basin is almost the same, whether it is outer or inner block. In addition, we reproduce high seismic activity in the Sagaing fault that validates the performance of the BAFD in the modelling of complex regions with various styles of faulting (Supporting information, Experiment 7).

Thus, the analysis of synthetic seismicity supports our choice of the preferred model. Assuming that all faults are locked, the preferred model is the best approximation to the observed tectonic motions and the major features of the recorded seismicity. This is also consistent with paleoseismic studies^15,37^. A fully or partially unlocked IBD has the maximum impact on simulation results, while the effect of the unlocked CMF is insignificant.

## Discussion

The BAFD modelling suggests the presence of a locked megathrust along the Indo-Burman arc. We came to this conclusion because the model fits the observed tectonic motions better, especially as regards the low velocity relative to India in the Tripura wedge, under the assumption of a locked megathrust. Assuming the unlocked IBD, the predicted velocities in the Tripura block concerning India are much higher than the observed ones (Figs. 2, 3). This conclusion is consistent with the results of other researchers. To be specific, Mallick et al.^3^ used a spherical model of elastic dislocations, horizontal strain rates, and block‐like motion to infer that the India and Burma plates are fully coupled to a depth of ~ 30 km and ~ 200 km east of the recent deformation front. Steckler et al.^4^ used GPS measurements of plate motions in Bangladesh combined with measurements from Myanmar and northeast India to suggest that the subduction at the Indo-Burman arc is still active and that there is a locked megathrust plate boundary despite the highly oblique plate convergence and the presence of thick sediments.

There is no reliable evidence about great earthquakes in Tripura. It was believed that a huge sedimentary system cannot produce the stresses and strain-weakening behavior needed for the occurrence of strong earthquakes^38^. Nevertheless, some of the largest known events have occurred in the thick sedimentary layer associated with the subduction zones^39,40^, such as the 1964 Alaska and the 1700 Cascadia earthquakes. A large, but poorly documented earthquake of 1548 is the only known candidate for a rupture of the plate boundary along the onshore part of the Indo-Burman collision zone^12^. Yet, poor-constrained location does not allow us to assign this earthquake to the Indo-Burma arc while Indian records report that the 1548 earthquake took place in Assam^41^, possibly on the Naga thrust. The most recent notable event in the Tripura occurred in 1918 with *M*w7.5 (the Centennial catalog^10^) at the northern end of the segment, but it cannot be unambiguously viewed as a subduction zone earthquake on the IBD. The present seismicity in Tripura is not high and occurs mainly on steep shallow strike-slip faults. However, the focal mechanisms of some earthquakes are consistent with a rupture along the gently dipping IBD^12,13^ (Fig. 1).

We modelled 12 great M8+ earthquakes in the Tripura section (Fig. 4) with an average recurrence time of about 1700 years. The maximum magnitude is modelled to be 8.3. These earthquakes occur irregularly up to 3 events per millennium, while inter-event intervals may reach 4 thousand years (Figure S11). As well, 50 large synthetic earthquakes have magnitudes between 7.5 and 8, i.e., our model generates about 3 large/great events per millennium in the Tripura region.

Cummins^16^ found some evidence for a high potential for giant earthquakes along the coast of Myanmar. This evidence is based on the similarity of the tectonic environment in the Indo-Burman Arc with those in other subduction zones of the world that experience giant earthquakes. Crustal stress and strain observations indicate that the seismogenic zone is locked and historical data on giant tsunami confirm the high potential of the Indo-Burman megathrust for generating great earthquakes. Historical records indicated that the 1762 earthquake caused extensive damage along the Arakan segment. A paleoseismic study of coral growth bands provided clear evidence of a coseismic uplift for the 1762 earthquake and two large earthquakes occurring in ~ 700 and ~ 1140 CE^37^. This work estimated the earthquake recurrence intervals of ~ 500 years. The study^15^ proposed that the 1762 earthquake has resulted from slip-on splay faults under the Cheduba and Ramree islands in addition to rupturing of the megathrust. The island’s uplift histories suggest recurrence intervals of such events of about 500–700 years. We modelled seven great earthquakes with *M* ≥ 8 in the Arakan section (Fig. 4) with much longer recurrence times of the order of 3000–5000 years and the maximum size of a synthetic earthquake in the Arakan section is modelled to be M8.4. Two great M8+ earthquakes occurred simultaneously on adjacent segments, which may be interpreted as multi-segment mega-earthquakes with magnitudes 8.7 and 8.6. Their rupture zones cover the southern part of the IBD (Fig. 4a). Such events may be similar to the 1762 Arakan earthquake (Fig. 1). The M8+ earthquakes revealed a near-periodic occurrence (Figure S11). Another 14 synthetic earthquakes with magnitudes between 7.5 and 8 have also been modelled in the Arakan section. Overall, the large events show irregular occurrence, up to 4 shocks per millennium, while the inter-event interval may reach 3 thousand years.

Several studies suggest that the India-Burma plate boundary is aseismic^17,18^ because none or insignificant subduction occurs across the Indo-Burman arc and the relative plate motion is accommodated in dextral strike-slip faults such as the CMF and Sagaing faults. Therefore, an aseismic slip in the CMF significantly reduces the seismic hazard of great interplate earthquakes^17^. The results of our modelling show a low coupling in the CMF that does not prevent great earthquakes from occurring in the IBD (experiments 1 and 5). Panda et al.^5^ suggested that the convergence across the blind megathrust (~ 7 mm/y) is significantly lower than that were estimated earlier. Due to the large scatter in the GPS data, it is not certain whether it is accommodated through creep or earthquakes. The estimate^5^ of the megathrust shortening of 7 mm/year is based on the relative motion of India and the undeformed Sundaland block located much south of the study area, rather than the Shan plateau. Authors excluded westward motion of the Shan plateau relative to Sunda at a velocity of ~ 10 to 12 mm/y that occur due to Tibetan crustal flow. Whether this is correct depends on the depth extent of the toroidal flow at Shan. Shi et al.^42^ showed that the model of deep asthenospheric flow around the Eastern Himalaya Syntax^43^ best explains the observed velocities and fault dynamics in the Shan Plateau between the Sagaing fault and Red River fault, while the upper crustal flow model meets some contradiction with observations^42^. Southeastern Tibetan crust is thought to be coupled to the mantle lithosphere, and thus, movement of the entire crust is likely the same as the uppermost mantle; this inference is supported by the mechanical coupling across the upper and lower crust^44^. In this case, the eastward motion of the Shan cannot be neglected. We infer that the IBD is locked, and rare great earthquakes release the slip deficit, which is 5–7 mm/y.

Our estimates of recurrence time for M8+ earthquakes are much longer than 500–800 years proposed in other studies^15,37^. The estimate^37^ is very preliminary as it is based on few dates, so it has to be taken as a minimum recurrence interval. A shorter recurrence period derived in^15,37^ is based on two assumptions: (i) all shortening across Burman arc is accommodated exclusively in the IBD; (ii) IBD is fully locked. Our result suggests that half of the shortening occurs in other faults, namely, in the Kabaw and the Kaladan (Fig. 3). These faults play important role in the complex regional geodynamics and strain portioning across the Indo-Burman arc, and this is supported by other studies. Mallick^3^ suggest that the Burma plate interface accommodates ~ 12 to 24 mm/year of oblique India‐Burma motion, which may further be partitioned among various upper plate structures in the seismic cycle. Steckler et al.^4^ showed that including the locked Kabaw fault in their model improves the reproduction of the GPS observations. Wang^15^ have found paleoseismic evidence of uplifts along the western coast of Cheduba and Ramree islands resulted from coseismic slip on splay faults, which are likely rooted in the megathrust. In our model, these splay faults may be associated with the southern segment of the Kaladan fault zone. These studies validate a contribution of the Kabaw and the Kaladan faults to the shortening across the Indo-Burman arc. This leads to an increase in the recurrence period of great earthquakes in the IBD. Perhaps, the BAFD overestimates the contribution of the Kabaw and the Kaladan to regional shortening due to the same rheology of faults. Using different rheology (more rigid Kabaw and Kaladan compared to IBD) can increase the portion of shortening in the detachment and reduce the recurrence time of major earthquakes in the IBD.

On the other hand, our result does not contradict paleoseismic studies^15,37^, since we get irregular occurrence of large events, up to 4 shocks with magnitude M7.5+ per millennium. This irregularity is explained by complex geodynamics and strain portioning due to oblique convergence of India and Burma plates causing clockwise rotation. In Himalaya, through the BAFD modelling, we explained why the seismicity in Bhutan differs from other segments of Himalaya with a similar structure and rate of shortening^24^. We showed a key role of the microplates Shillong plateau and Assam basin: their motion relative to the Indian plate causes an increased rate of rotation in Bhutan and leads to a decrease in seismic activity and maximum magnitude, and an irregular occurrence of large earthquakes. Additionally, there is a lack of GPS observations in the Arakan section (Fig. 1). New geodetic measurements can refine model parameters and lead to a reevaluation of the recurrence period of great earthquakes.

Mallick et al.^3^ and Steckler et al.^4^ inferred that all faults included in their models are fully coupled. We obtained high coupling in the IBD, Kaladan, and Kabaw faults, while in the central segments of CMF coupling is low (Figure S9; Table S6). The modelled coupling in the IBD increases southward from 0.6 in the north of Tripura to 0.9 in the south of the Arakan segment. The difference in the seismicity patterns simulated in the Arakan and Tripura is consistent with the results of the study^45^ that explained the variation of rupture characteristics in subduction zones by the level of coupling between interacting tectonic plates. Strong-coupled zones produce rare periodic mega-earthquakes, while moderate-coupled segments produce more numerous large shocks that tend to cluster over time, but mega-earthquakes are less probable. Similar patterns were observed in Kamchatka: the southern segment hosting the source of the 1952 Kamchatka catastrophic earthquake of *M* = 9.0 has been identified as a strong-coupled zone, whereas northern Kamchatka is a moderate-coupled zone where several large earthquakes with magnitudes ≈8 have been recorded^46^.

## Conclusions

Recent GPS measurements in Bangladesh, Myanmar, and northeast India indicate 13–17 mm/y of plate convergence along a low-angle dipping megathrust. How is the relative plate motion released in this very large active fault? That is a critical issue for seismic hazard assessment in this densely populated region. Possibilities include a broad range of patterns from continuous aseismic creep to very large and relatively rare earthquakes, or all patterns occurring on different portions of the megathrust^3,12^. The lack of a clear historic precedent of great seismic events, the thick over-pressured sediments of the Ganges–Brahmaputra delta (GBD), and the very oblique convergence of India and Burma plates made researchers lean toward the aseismic option^17,18^. Nevertheless, several recent studies^3,4^ based on new GPS observations suggest a locked Indo-Burman megathrust and possible great earthquakes.

In the present work, using the BAFD model, we have tested the following two hypotheses:

(i) Is the India-Burma detachment is locked?
(ii) Is the detachment able to produce great earthquakes?

We have shown that the model of locked detachment is preferred because it more adequately reproduces observed tectonic velocities. The advantage and novelty of our study is a 20 thousand years long synthetic earthquake catalogue, which allows estimating the maximum magnitude and recurrence period of great shocks in the Indo-Burman megathrust. The integral characteristics of synthetic seismicity, the earthquake size distribution and the rate of seismic activity are compatible with those derived from the observed seismicity, historical records and paleoseismic studies^15,37^. Our results suggest that the megathrust is locked and can generate great M8+ earthquakes with a long recurrence period exceeding 1000 years. We modelled two mega-earthquakes with magnitudes 8.7 and 8.6 in the southern section of the Indo-Burman arc, which may be similar to the 1762 Arakan earthquake. This is supported by paleoseismic studies^15,37^. Additionally, we obtained several M8+ events in the northern onshore section of the megathrust where no great earthquakes have been reported by the instrumental and historical records.

Our results suggest that the Kaladan and the Kabaw faults are active locked faults playing a significant role in the regional dynamics and seismicity. They accommodate about half shortening across the Burman arc. This explains the increased recurrence time of great earthquakes in the IBD. The properties of the CMF are unresolved since they give little impact on the results of the modelling. The CMF is primarily strike-slip and almost does not contribute to regional shortening therefore it does not affect seismicity in the IBD. The CMF appears to be sliding rather than locked because we have estimated a low coupling in this fault.

The BAFD model does not aim to reproduce observations in all their details since it assumes a very simplified description of the study region. Particularly, we did not include into the block-structure small steep strike-slip faults in Tripura or many small splay faults of the foldbelt above the IBD. The BAFD model does not include fluids in the subducted GBD sediments, which may affect the style of stress release in the detachment. There is also a lack of GPS data in the south of the study region.

Despite the above shortcomings, several studies have confirmed the performance of the BAFD model. Modelling the Tibet-Himalaya region Ismail-Zadeh et al.^26^ have predicted a cluster of large earthquakes in Eastern Sichuan, where no such events were known at that moment. In 2008, the *M*7.9 Sichuan earthquake validated the modelling results. For different sections of the Himalayan arc, Vorobieva et al.^24^ reproduced seismic cycles varying from 700 to 2100 years, which agrees with the results of recent paleoseismic studies^47,48^.

Based on the BAFD modelling, we inferred the presence of a locked megathrust along the Indo-Burman arc and the potential of future great earthquakes whose effect can involve millions of people in Bangladesh, Myanmar and Northern India. Our results contribute to regional seismic hazard and risk assessment.

## Methods

### Basic principles of the BAFD model

The BAFD model^19–21^ was designed based on the hypothesis of the block structure of the Earth’s lithosphere^49^. Numerous GPS observations and models, from regional ones^32^ to global^50^, support the concept of the Earth’s crust as a system of rigid blocks separated by narrow deformation zones. The concentration of deformation in the narrow zones separating the blocks from each other is justified by the fact that the effective elastic modulus in the fault zones is significantly less than those within the blocks^51,52^, and the viscosity is 2–3 orders less^53,54^. Thus, the strain in the fault zones is 3–4 orders higher than within blocks and allows blocks to be considered as rigid.

We assume that a seismic region is a structure of rigid crustal blocks. Blocks are separated by infinitely thin visco-elastic faults, which may have arbitrary dip angles (Figure S1). Blocks interact with each other and with the lower crust and move in response to a prescribed tectonic motion at the lateral boundaries, and to basal motions. The visco-elastic interaction is described by the Maxwell rheological law for the stress and strain relation^19^ (Equations S1-S4). The stress is proportional to the strain, and the rate of viscous deformation is proportional to the stress (Equation S4).

The motion of blocks (shift and rotation) is updated at each time step of numerical simulation. First, we calculate inelastic displacement at each cell by the current shear stress in the faults. Second, we update positions of external blocks and lower crust according to specified velocities. The elastic forces arise in fault planes and block bottoms in response to these external motions. Next, new positions of the inner blocks are calculated. The shift vectors and the angles of rotation of blocks are determined by the condition that the structure is in a quasi-static equilibrium. That is the sum of forces and moments of forces, acting on each block must be zero.

Earthquakes are simulated according to the Coulomb failure stress criterion and the dry friction model (Equations S5, S6). Thus, when the ratio of the shear stress to the difference between the normal stress and the lithostatic-hydrostatic pressure exceeds the friction coefficient, instant co-seismic slip occurs. The size of the earthquake is the area of the ruptured portion of the fault, which could be converted to magnitude using classical empirical formulas^55^ (Equation S7). The earthquakes are allowed in the faults but restricted in the block bottoms. Earthquakes and slow motions are simulated simultaneously in one run of the BAFD model.

The BAFD model is designed to simulate dynamics and seismicity of the upper crust in the regions where tectonics plays a major role. It is not suitable to model non-tectonic seismic regions, such as zones of volcanic, thermal or induced seismicity.

### Input data and output of the BAFD model

The following data should be introduced to simulate tectonic motions and earthquakes in the BAFD model:

(i) The geometry of crustal blocks is outlined based on the mapped faults, morpho-structural zonation, seismotectonic models, etc. (Fig. S4). The block’s depth and the dip angle of each fault are chosen based on any available information about the deep structure of the region.
(ii) The external tectonic velocities at the lateral boundaries of the block structure are prescribed by GPS measurements (Table S1). The basal velocities at the bottom of the blocks are chosen based on any available information about the deep structure of the region and GPS velocities on the surface (Table S2).
(iii) Rheological (elastic and viscous) parameters of faults and bottom of blocks. As faults are infinitely thin, the rheological parameters describe elasticity and factor for the rate of inelastic displacements in the entire faults zone.
(iv) The parameters for earthquake occurrences: friction coefficient, the difference of lithostatic and hydrostatic pressure, and the stress drop due to earthquake (Table S4).
(v) Period, time step, and the size of the cell discretizing fault segments.

No additional inputs, like the style of faulting, seismic coupling, etc., are required for the BAFD modelling.

The primary outputs of modelling are the block motions including rotation, and the earthquake catalogue, where each event has origin time, hypocenter, magnitude and focal mechanism. Then we can derive interseismic velocities of blocks (velocity field), relative motions in the faults, seismic coupling, etc.

The input parameters for the modelling cannot be determined uniquely since the observations are incomplete, have limited accuracy, and are consistent with different interpretations. With varying parameters, the result of modelling may change. From the previous experience in the BAFD modelling, we know that small changes in the input parameters do not lead to significant changes in the result. We note that synthetic seismicity is more sensitive to input parameters than block motions.

Despite the simplicity based on principles, the dynamics of the block structure demonstrate the complex non-linear behavior (Equation S8). The state of a particular fault segment depends not only on its dynamics and seismic history but also on the dynamics of all elements of the block structure. Numerical experiments described in the present study demonstrate the non-local effect of rheological parameter changes in specific faults. The same changes of a particular parameter may lead to the opposite effect depending on the other parameters. For example, with the increase in fragmentation of the fault network the maximum magnitude decreases in the case of contraction but increases in the case of rotation^56^. Therefore, due to the non-linear behavior of the model, non-local effects, cross-influence of the different input parameters the influence of a particular parameter on the result may be different (or even opposite) depending on the block structure geometry and type of movement. All these factors can be determined only by numerical experiments^22–24^.

### Evaluation of modelling results

We evaluate modelling results by comparing them with available observations, such as GPS measurements and earthquake catalogues. The period of instrumental recording of seismicity is usually several decades that is much shorter than the recurrence time of large earthquakes. The long-term characteristics of seismicity, e.g. activity, maximum magnitudes and some others may differ from those instrumentally detected nowadays. That makes essential the use of historical records and paleoseismic studies to accept or reject modelling results^24^, this study_._

### Interpretation of model assumptions

#### Rigid blocks and elastic forces in the infinitely thin faults

The change of strain in the block structure is very small at each time step of simulation: with the size of region ~ 10^6^ m, the tectonic velocities ~ 10^−2^ m/year, and the time step ~ 10^–2^ year, the strain change is $10^{ - 10} - 10^{ - 9}$. Therefore, we neglect the changes in the size and shape of blocks at each particular time step. We regard them as rigid bodies, the kinematics of which is reduced to a shift and rotation. From the viewpoint of dynamics, the system of blocks has viscous-elastic properties, and the elastic stress arises in response to displacement. We just attribute equivalent viscous-elastic properties to infinitely thin layers separating blocks (model faults). Still, deformations are small, the same force $\vec{F}$ arises in response to the identical shift *dL* whatever is an internal structure of the blocks (Figure S2).

#### Fixed geometry and rate of the external tectonic motions

The model is designed to simulate dynamics and seismicity in short periods compared to geological times (up to tens of thousands of years) when the regional fault network does not change. Typically, the total strain during simulation must be less than 10^–3^. We assume the stationary tectonic velocities since there is no reliable information about changes due to a short period of GPS observation.

#### Model faults and seismicity

Synthetic earthquakes are simulated in the model faults, while the spread seismicity is typical for many seismic regions, and little instrumental earthquakes can be attributed to a particular large fault included in the block structure. In the BAFD model, a single fault represents a strain accumulation zone that has a width up to tens of kilometers and the complex structure including, in addition to the main fault, a plurality of small seismogenic faults. When we evaluate synthetic seismicity, we assign all earthquakes that occurred in the region to the model fault zones.

#### Dip structure

The model does not consider the heterogeneity that may present at depths, i.e. all faults have the same depth, and the rheology does not change with depth. The average values are used for the entire fault segment in the model.

#### Gravity

The gravity is not included in the BAFD model, and the forces driving blocks arise only due to external motions. However, the use of basal motions allows simulation in a simplified form of the negative buoyancy of the subducting plate, and uprising flows beneath the upper plate as shown in Figure S3.

Further details and governing equations of the BAFD model are provided in the Supporting information.

## Supplementary Information

Supplementary Information.

## Data Availability

The ANSS Earthquake catalog https://earthquake.usgs.gov/data/comcat/; the Global Centroid-Moment-Tensor Catalog https://www.globalcmt.org. Other data are listed in the References.
