# Supplementary material for: Modelling the seismic potential of the Indo-Burman megathrust
Source: Sci Rep. 2021 Oct 27;11:21200. doi: 10.1038/s41598-021-00586-y (PMC8551320; doi:10.1038/s41598-021-00586-y)
Supplement: Supplementary file 1 — Supplementary Information. [file 41598_2021_586_MOESM1_ESM.pdf]

Supporting information for

Is the Indo-Burman megathrust capable of generating great earthquakes?

Results from the Block-and-Fault Dynamics Modeling

Inessa Vorobieva[1], Alexander Gorshkov[1], Prantik Mandal[2]

[1]Institute of Earthquake Prediction Theory and Mathematical Geophysics, Russian Academy of Sciences, 84/32 Profsouznaya, Moscow 117997, Russia

[2]CSIR-National Geophysical Research Institute, Uppal Road, Hyderabad-500007, A.P., India. Email: prantik@ngri.res.in

Table of contents

1 Description of the BAFD model and governing equations p2

2 GPS measurements. p6

3 Geometry of the block structure of the Indo-Burman arc and numerical parameters of modeling p8

4 Details of numerical experiments p10

4.1 The block motions as modeled in six experiments p10

4.2 Coupling in the preferred experiment p13

4.3 Temporal sequences of earthquakes in the IBD p15

4.4 Synthetic seismicity modeled in the experiments 2-6 p16

5 Experiment 7 “Including the Sagaing fault” p22

5.1 The block motions p22

5.2 Synthetic seismicity p23

1. Description of the BAFD Model and Governing equations

The BAFD model considers a seismic region as a structure of rigid crustal blocks separated by infinitely thin visco-elastic faults. The block-and-fault structure is a bounded and simply connected part of a layer of thickness *H* limited by two horizontal planes. The lateral boundaries of the structure and its subdivision into blocks are formed by portions of fault planes intersecting the layer. The fault planes may have arbitrary dip angles, which are specified basing on the knowledge of the deep structure of the region (Figure 1a). Each fault segment must completely intersect layer from top to bottom of block structure. Figure S1b, c illustrates allowed and restricted fault geometry.

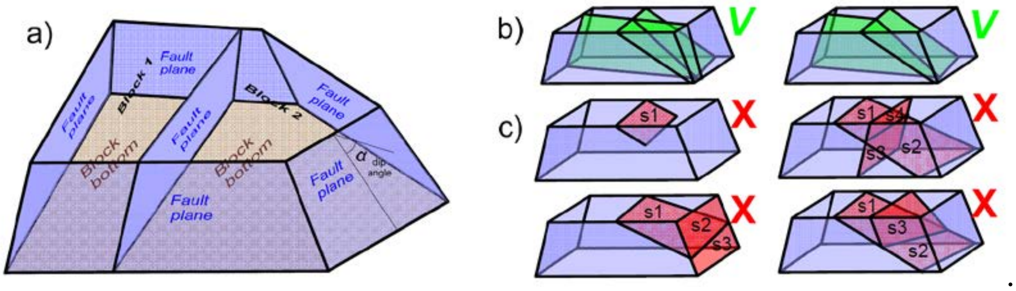

Fig. S1. Geometry and elements of a block structure for the BAFD modeling. **a)** Elements of the block structure. **b)** Allowed fault geometry. **c**) Restricted fault geometry: red fault segments **s1**…**s4** do not completely intersect the layer.

The blocks interact each other and with the lower crust, and move in response to prescribed motion of the confining medium and of the underlying medium. The interaction is visco-elastic. The external motion is defined in the segments of lateral boundary (boundary blocks). The basal motion of the lower crust is prescribed on the block bottoms. The movement rates of the underlying medium and of the boundary blocks are assumed to be horizontal and known, and can be different for each block.

The relative displacements of the blocks take place along the fault planes. Elastic forces arise in the lower plane and in the fault planes in response to the displacement of the blocks relative to the underlying medium, to the lateral boundary, and to the other blocks. The vector of elastic shear stress $\boldsymbol{\sigma}$ at point (*X*, *Y*) of the fault plane, separating two blocks, is calculated as:

$$\boldsymbol{\sigma} = K(\boldsymbol{\Delta} - \boldsymbol{\delta}) \quad \text{(S1)}$$

where $\boldsymbol{\Delta}$ is vector of total relative displacement and $\boldsymbol{\delta}$ is vector of the inelastic slippage at the point (*X*, *Y*) of the fault plane. The rate of inelastic slippage is proportional to the shear stress $\boldsymbol{\sigma}$:

$$\frac{d\boldsymbol{\delta}}{dt} = W\boldsymbol{\sigma} \quad \text{(S2)}$$

The same equations (S1, S2) govern the interaction of blocks with the lower crust.

The equations (S1) and (S2) describe the Maxwell rheological law for the stress $\boldsymbol{\sigma}$ and strain $\boldsymbol{\zeta}$ relation:

$$\left[\frac{d}{dt}+\frac{1}{\tau}\right]\boldsymbol{\sigma}=\mu\frac{d\boldsymbol{\zeta}}{dt};\ \tau=\frac{\eta}{\mu} \tag{S3}$$

Here μ,η and τ represent elastic shear modulus, viscosity, and relaxation time, respectively. The coefficients in equations (S1), (S2) and (S3) are connected through following relations:

$$K=\frac{\mu}{a};\ W=\frac{a}{\eta} \tag{S4}$$

Here 'a' is the width of the deforming zone, and *K* and *W* are related to one unit of the model time *t*. In Equations (S1, S2), $K=\mu/a$ describe the elasticity of the entire fault zone. It is measured in bar/cm, or 10^7 Pa m^{-1}. Similarly, the $W=a/\eta$ is the factor for the rate of inelastic displacements in the entire fault zone. It is measured in cm bar^{-1}Unit Model $Time^{-1}$, or 10^{-7} m Pa^{-1}Unit Model $Time^{-1}$. The unit of model time may be different depending on the regional tectonic velocities, and usually it is 1 year or 10 years, sometimes, with very small tectonic velocities, it may be 100 years. We set the value of the unit of model time when we prescribe the rate of external motions. This technique allows minimizing numerical instability.

On the fault plane, the reaction force is normal to the fault plane and its size, per unit area, is:

$$\sigma_{\mathrm{n}}=\sigma_{\mathrm{l}}\tan\alpha \tag{$S5$}$$

where σ_{l} is the component of the elastic shear stress $\boldsymbol{\sigma}$ normal to the fault line on the upper plane, and α is the dip angle of the fault plane. Therefore, total stress is horizontal. The value of σ_{n} is positive in the case of extension and negative in the case of contraction.

The displacements of the blocks and are presumed to be infinitely small, compared with the block size. Therefore, we neglect the changes in the size and shape of blocks at each time step of modeling. We regard them as rigid bodies, the kinematics of which is reduced to a shift and rotation around their geometrical centers. At each time point the displacements of the blocks are found from the condition that the total force and the total moment of forces acting on each block are equal to zero. This is the condition of quasi-static equilibrium of the system and, at the same time, the condition of minimum energy. The equilibrium equations include only forces caused by the specified basal and external movements. The action of all other forces (gravity, etc.) on the block-structure is ruled out and does not cause displacements of blocks.

The space discretization is necessary to carry out the numerical simulation of block-structure dynamics. Fault planes and block bottoms are divided into cells of a given linear size. The relative displacement $\mathbf{\Delta}$, the inelastic slippage $\boldsymbol{\delta}$, and the elastic stress $\boldsymbol{\sigma}$ are assumed to be constant within a cell. We calculate a state of the block structure at discrete times $t_i = t_0 + i\Delta t$, where t_0 is the initial time. The transition from the state at t_i to the state at t_{i+1} proceeds as follows:

(i) new values of the inelastic displacements in the each cell of the fault planes and block bottoms are calculated accordingly to current stress $\boldsymbol{\sigma}$ at the moment t_i using equation (S2);

(ii) positions of external blocks and lower crust are updated according to specified velocities, and new vectors of elastic shear stress $\boldsymbol{\sigma}$ are calculated at time t_{i+1} using equation (S1);

(iii) The new shift vectors and the angles of rotation of blocks are determined by the condition that the structure is in a quasi-static equilibrium.

Earthquakes are simulated according to the Coulomb failure stress criterion and the dry friction model. For each cell of the faults we calculate value of κ:

$$\kappa = \frac{|\boldsymbol{\sigma}|}{P - \sigma_{\mathrm{n}}} \qquad (S6)$$

where $|\boldsymbol{\sigma}|$ is magnitude of the shear stress, σ_n is the normal stress, and *P* is the difference between the lithostatic (due to gravity) and the hydrostatic pressure. If the value of κ exceeds the prescribed friction coefficient *B* in a cell, an abrupt slip $\boldsymbol{\delta}^{\mathrm{e}}$ occurs in this cell to reduce the value of κ to the given level H_{f}. The slip $\boldsymbol{\delta}^{\mathrm{e}}$ occurs in the fault plane in the direction of the shear stress vector $\boldsymbol{\sigma}$**.** Once the new values of the inelastic displacements for all the failed cells are computed, the translation vectors and the angles of rotation of the blocks are determined to satisfy the condition of quasi-static equilibrium. As a result, κ may exceed the friction coefficient *B* in other cells. The procedure is repeated until κ falls below *B* in all cell of all faults. At the moment t_i, each connected cluster of the ruptured cells form a single earthquake. The size of the earthquake is the area *S* of the ruptured portion of the fault, which could be converted to magnitude using classical empirical formulas (Wells and Coppersmith, 1994).

$$M\mathrm{w} = 0.98 \cdot \log_{10} S + 4.07 \qquad (S7)$$

The area *S* is measured in km^2.

For each earthquake, the source mechanism can be determined. The strike and dip angles are prescribed by the block structure geometry, and the rake is the direction of slippage $\boldsymbol{\delta}^{\mathrm{e}}$ in the fault plane.

In the classical BAFD model, the effective viscosity (and hence W) of the fault zone is constant. In the present study, we use a nonlinear rate-dependent slip at the faults, that is, the rheological parameter W (or the effective viscosity) depends on the rate of inelastic displacement $\dot{\delta}$ expressed in the form:

$$W = W_0 \left(1 + \varepsilon \dot{\delta}^{1/2}\right) \quad \text{(S8)}$$

Where, W_0 corresponds to the linear viscosity, and $\dot{\delta}$ is the rare of inelastic displacement in the preceding time moment. The value of the constant ε assumed to be small, so that in the interseismic periods $W \approx W_0$, and almost does not depend on $\dot{\delta}$. At the moment of an earthquake, the rate $\dot{\delta}$ increases abruptly in the broken cell. This leads to the growth of the rate of inelastic parameter W. Then, as $\dot{\delta}$ decreases, the value of W decays gradually to W_0, and the ruptured cell returns to the normal state.

The following data should be introduced to simulate tectonic motions and earthquakes in the BAFD model:

(i) The geometry of crustal blocks outlined based on the mapped faults, morpho-structural zonation, seismotectonic models, etc. The block's depth, and the dip angle of each fault is chosen basing on any available information about deep structure of region.

(ii) The external tectonic velocities at the lateral boundaries of the block structure are prescribed in accordance with GPS measurements. The basal velocities at the bottom of the blocks are chosen based on any available information about deep structure of region and GPS velocities in the surface.

(iii) Rheological (elastic and viscous) parameters K and W of fault segments and bottom of blocks. The K and W are constant within each segment, but may be different in different segment and at the block bottoms.

(iv) The parameters for earthquake occurrences: friction coefficient B, difference of lithostatic and hydrostatic pressure P, and the stress drop due earthquake H_f.

(v) Time period, time step, and the size of the cell discretizing fault segments.

The primary outputs of modeling are the block motions including rotation and the earthquake catalog where each event has origin time, hypocenter, magnitude and focal mechanism. Then we can derive interseismic velocities of blocks (velocity field), relative motions in the faults, seismic coupling, etc.

Interpretation of model assumptions.

Rigid blocks and elastic forces in the infinitely thin faults. The change of strain in the block structure is very small at each time step of simulation: with the size of region ~10^6m, the tectonic velocities ~10^{-2}m/year, and the time step ~10^{-2} year, the strain change is $10^{-10} - 10^{-9}$. Therefore, we neglect the changes in the size and shape of blocks at each particular time step. We regard them as rigid bodies, the kinematics of which is reduced to a shift and rotation. From the viewpoint of dynamics, the system of blocks has viscous-elastic properties, and the elastic stress arises in response to displacement. We just attribute equivalent viscous-elastic properties to infinitely thin layers separating blocks (model faults). Still, deformations are small, the same force $\vec{F}$ arises in response to the identical shift *dL* whatever is an internal structure of the blocks (Figure S2).

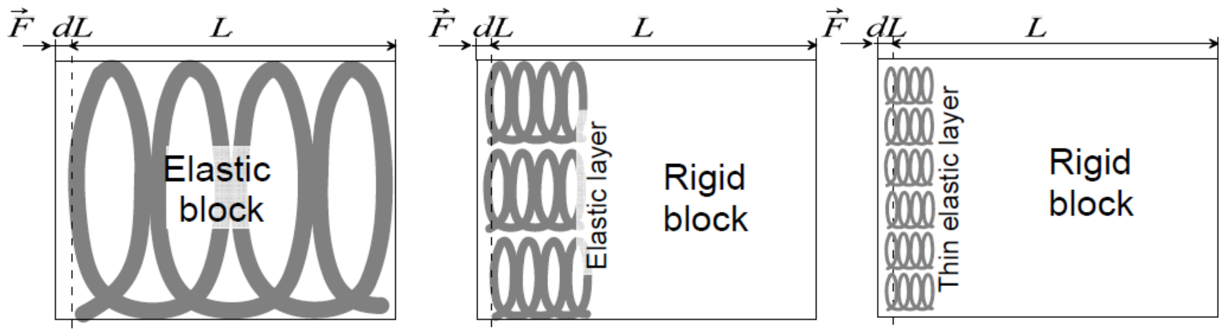

Fig. S2. Interpretation of the visco-elastic features in the structure of rigid blocks separated by infinitely thin faults.

Fixed geometry and rate of the external tectonic motions. The model is designed to simulate dynamics and seismicity in short periods compared to geological times (up to tens of thousands of years) when the regional fault network does not change. Typically, the total strain during simulation must be less than 10^{-3}. We assume the stationary tectonic velocities since there is no reliable information about changes due to a short period of GPS observation.

Model faults and seismicity. Synthetic earthquakes are simulated in the model faults, while the spread seismicity is typical for many seismic regions, and little instrumental earthquakes can be attributed to a particular large fault included in the block structure. In the BAFD model, a single fault represents a strain accumulation zone that has a width up to tens of kilometers and the complex structure including, in addition to the main fault, a plurality of small seismogenic faults. When we evaluate synthetic seismicity, we assign all earthquakes that occurred in the region to the model fault zones.

Dip structure. The model does not take into account the heterogeneity that may present at depths, i.e. all faults have the same depth, and the rheology does not change with depth. The average values are used for the entire fault segment in the model.

Gravity. The gravity is not included into the BAFD model, and the forces driving blocks arise only due to external motions. However, the use of basal motions allows simulation in a simplified form of the negative buoyancy of the subducting plate, and uprising flows beneath the upper plate as shown in figure S3.

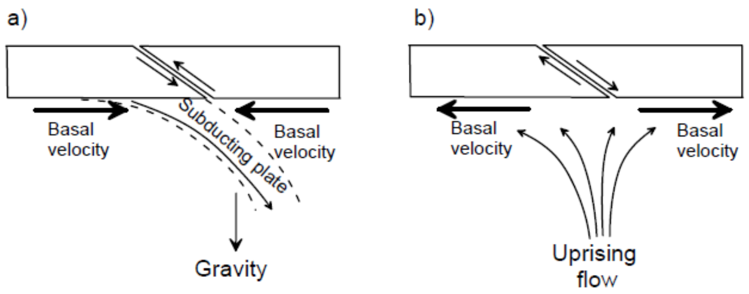

Fig. S3. Use the basal velocities in the BAFD for modeling of (a) negative buoyancy of the subducting plate, and (b) uprising flow in the asthenosphere beneath the upper plate.

2. GPS measurements.

We use one measurement for each site. Preferred sources in order of priority[1-6]: All velocities are in ITRF2008, relative to stable India. B1-B3 are inner blocks of the model, BB1-BB5 are external boundary blocks (Figure S1)

Table S1. List of GPS measurements

Lon °	Lat °	*Ve* (mm/yr)	*Vn* (mm/yr)	Name	Ref	Model block
**Velocities used to compare observations and model**						
92.19	23.16	-5.1	-3.0	BAGH	Steckler	B1
92.38	22.73	-5.2	-6.1	BARK	Steckler	B1
91.79	22.47	-1.4	-2.7	CHIT	Steckler	B1
91.52	24.19	-1.3	-2.7	CHNR	Steckler	B1
92.07	23.24	-5.0	-3.5	DIGH	Steckler	B1
92.02	25.18	-1.0	-5.9	JAFL	Steckler	B1
91.24	25.00	-0.3	-4.4	JAML	Steckler	B1
92.14	24.50	-4.7	-4.1	JURI	Steckler	B1
92.22	22.50	-4.8	-3.3	KPTI	Steckler	B1
91.85	22.85	-1.9	-0.3	MKCH	Steckler	B1
90.76	23.04	-0.2	-0.2	RPUR	Steckler	B1
91.66	22.62	0.9	-2.8	SITA	Steckler	B1
91.84	24.93	0.8	-8.8	SLET	Mallick	B1
91.73	24.31	-0.6	-6.0	SRIM	Mallick	B1
91.83	24.92	2.1	-5.0	SUST	Steckler	B1
92.49	23.94	-6.9	-4.4	MAMT	Gahalaut	B2
93.56	24.82	-5.4	-8.6	AWNG	Gahalaut	B2
92.73	23.72	-6.0	-6.6	AZWL	Steckler	B2
92.69	23.31	-8.1	-4.7	BRPI	Gahalaut	B2
92.90	23.18	-7.8	-5.9	BTLG	Gahalaut	B2
93.31	23.48	-8.7	-7.5	CHAM	Gahalaut	B2
92.73	23.76	-6.1	-8.0	DRNG	Mallick	B2
93.13	24.80	-5.5	-6.4	JIRI	Gahalaut	B2
92.87	24.81	-4.1	-5.8	KASH	Gahalaut	B2
93.09	23.61	-8.8	-5.9	KKHL	Gahalaut	B2
92.73	24.37	-6.6	-4.6	KLSB	Gahalaut	B2
93.08	23.20	-9.1	-5.9	LGDR	Gahalaut	B2
93.06	23.13	-9.2	-6.5	NVPI	Gahalaut	B2
92.86	23.33	-6.7	-5.0	SRCP	Gahalaut	B2
93.68	24.34	-11.0	-14.3	CCPR	Gahalaut	B3
94.01	24.36	-12.5	-14.9	CHAN	Gahalaut	B3
94.51	24.868	-12.1	-16.0	CHAS	Gahalaut	B3
93.60	22.63	-12.8	-16.2	HAKA	Mallick	B3
93.93	24.75	-11.4	-14.8	IMPH	Gahalaut	B3
93.79	24.82	-9.7	-11.6	KJRK	Gahalaut	B3

93.90	21.383	-9.8	-13.9	MIND	Maurin	B3
94.29	24.26	-12.8	-16.2	MORE	Gahalaut	B3
94.15	24.38	-11.6	-16.1	TENG	Gahalaut	B3
Velocities used to prescribe external motions						
90.40	23.73	-0.9	0.1	DHAK	Steckler	BB1
90.34	25.12	1.0	-2.1	HGAT	Mallick	BB1
88.74	22.90	2.9	-1.5	ICHA	Mallick	BB1
89.54	22.80	0.6	-0.1	KHUL	Steckler	BB1
89.53	22.85	2.0	-3.0	KLNA	Mallick	BB1
90.03	24.60	0.8	-2.3	MPUR	Steckler	BB1
90.24	22.36	0.3	-1.4	PUST	Steckler	BB1
88.64	24.37	-1.5	-0.4	RAJS	Steckler	BB1
90.40	24.41	3.0	-5.5	VLKA	Mallick	BB1
91.69	25.27	0.6	-7.7	CRPJ	Mallick	BB2
91.86	25.567	-0.4	-5.6	CSOS	Vrenant	BB2
91.57	25.699	-2.4	-1.9	MAWP	Vrenant	BB2
91.44	25.233	-0.2	-2.6	MOPE	Vrenant	BB2
91.83	25.56	-1.1	-8.8	MTWR	Mallick	BB2
91.84	25.413	0.2	-5.5	MUNN	Vrenant	BB2
91.86	25.9	-0.5	-5.3	NONG	Vrenant	BB2
91.44	25.23	-0.5	-3.3	NOPE	Banerjee	BB2
91.11	25.668	2.2	-4.7	PORL	Vrenant	BB2
91.72	25.256	0.6	-2.2	RANG	Vrenant	BB2
91.89	25.566	1.1	-4.1	SHLN	Banerjee	BB2
94.20	25.652	-4.9	-11.2	CHAK	Gahalaut	BB3
94.01	25.261	-9.9	-13.0	SENA	Gahalaut	BB3
94.36	25.109	-10.3	-16.1	UKHR	Gahalaut	BB3
94.04	25.718	-3.1	-10.7	ZUBU	Gahalaut	BB3
95.92	21.672	-17.8	-25.1	CHAU	Maurin	BB4
95.60	21.962	-15.9	-21.5	HTIS	Maurin	BB4
95.87	23.80	-19.6	-22.4	INND	Steckler	BB4
94.85	22.43	-13.3	-20.6	KANI	Mallick	BB4
95.29	22.049	-17.8	-21.2	KWEH	Maurin	BB4
95.76	21.986	-16.1	-24.5	LEGY	Maurin	BB4
95.72	21.691	-16.5	-22.6	MYOT	Maurin	BB4
95.92	21.991	-13.6	-24.8	SAYE	Maurin	BB4
95.72	22.57	-14.1	-17.1	SWBO	Mallick	BB4
95.81	22.162	-16.8	-23.0	THIT	Maurin	BB4
95.78	22.367	-16.8	-26.8	WETL	Maurin	BB4
95.75	23.85	-16.3	-21.7	WUNT	Steckler	BB4
94.54	17.692	-7.3	-10.2	LAUN	Maurin	BB5

3. Geometry of the block structure of the Indo-Burman arc and numerical parameters of modeling

Our model includes the India-Burma Detachment (IBD), the Kaladan fault, the Churachandpur-Mao fault (CMF), and the Kabaw fault. The Dauki thrust limits the block structure in the north. In the south, the simplified EW boundary goes along the latitude of 18°N and separates the modelled area from the Andaman subduction zone. This boundary does not correspond a mapped fault zone.

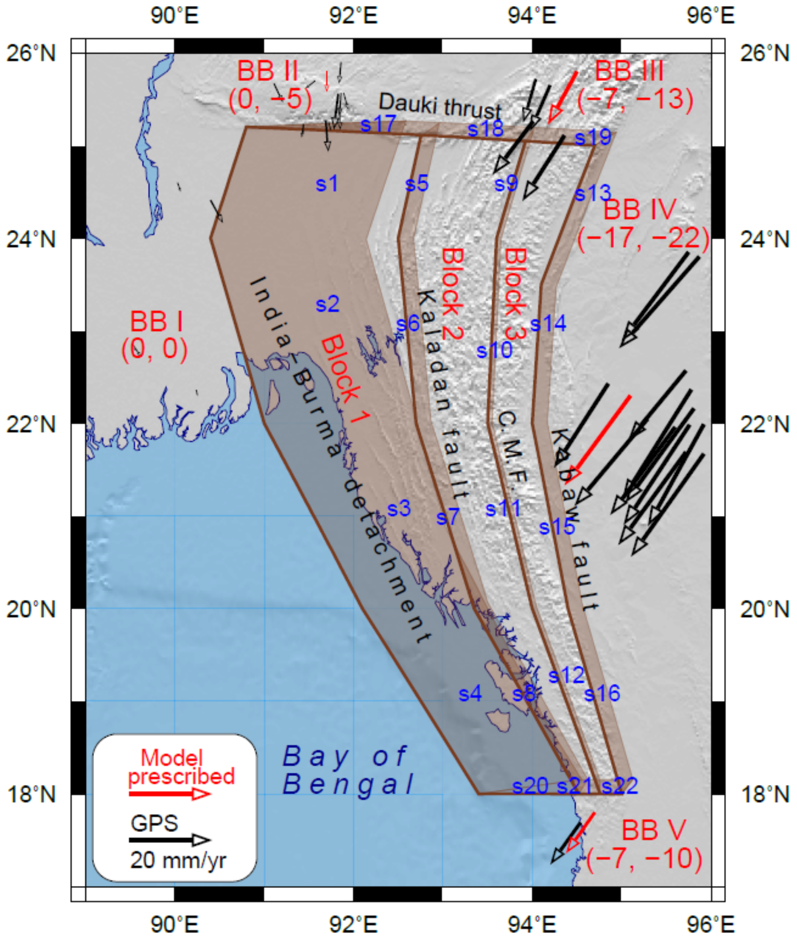

Fig. S4. Geometry of the block structure of the Indo-Burman arc and prescribed external velocities. GPS observations and prescribed external velocities are shown by black and red arrows, respectively. B1-B3 are blocks; BBI - BBV are boundary blocks. Each fault is outlined by several segments, s1-s22 are segment numbers. Fault geometry at depth is shown by transparent brown.

The extension of locked portion of IBD roughly coincide with the location of the Kaladan fault (Steckler et al., 2016, Mallick et al., 2019).

Table S2. External and basal velocities

**Block**	***V*x**, (North) mm/yr	***V*y**, (East) mm/yr	***ω***, angular velocity, 10^{-6} rad/yr
BB I, India plate	0	0	0
BB II, Shillong	0	-5	0
BB III, Assam	-7	-13	0
BB IV, Myanmar Basin	-17	-22	0
BB V, Sothern boundary	-7	-10	0
Bottom of Block 1	0	0	0
Bottom of Block 2	0	0	0
Bottom of Block 3	-17	-22	0

Table S3. Dip angles of faults

**Fault**		**Dip angle, º**
India-Burma detachment (IBD),	s1	10°
	s2	10°
	s3	13°
	s4	15°
Kaladan		60°
C.M.F		80°
Kabaw		60°
Dauki		60°
South		60°

Table S4. Numerical model parameters in formulas S1-S6

**Parameter***	**Value**
Time span of modeling	20000 year
Time step	0.04 year
Space discretization	2 km
Unit Model Time	1 y
K**, elastic coefficient for the faults and block bottoms	1 bar/cm
W_0***, rate of inelastic displacement	
Locked faults	$2{\cdot}10^{-4}$cm bar^{-1}year^{-1}
Unlocked faults	$2{\cdot}10^{-3}$cm bar^{-1}year^{-1}
Block bottoms	$2{\cdot}10^{-3}$cm bar^{-1}year^{-1}
P, difference of lithostatic and hydrostatic pressure	3000 bar
B, friction coefficient	0.5
H_f, earthquake stress drop	0.3

*Notation of parameters as in “Model description”, equations S1, S2, S6, S8

**With a width of deforming zone a≈10 km=10^6cm, and K=1bar/cm, the shear modulus

$\mu = Ka \approx 10^6\text{bar}{\approx}10^{11}\text{Pa}$ (see Eq S4 in “Model description”).

***Unit of model time is 1year≈$3{\cdot}10^7$sec, then with W=$2{\cdot}10^{-4}$cm bar^{-1}year^{-1} viscosity in locked faults $\eta = \frac{a}{W} \approx 5 \cdot 10^9 \text{bar} \cdot \text{year} \approx 1.5 \cdot 10^{22}\text{Pa} \cdot \text{sec}$, and in unlocked faults $1.5 \cdot 10^{21}\text{Pa} \cdot \text{sec}$

4. Details of numerical experiments.

List of experiments:

1. All faults locked (preferred experiment);
2. IBD unlocked;
3. Tripura section of IBD unlocked;
4. Kaladan unlocked;
5. CMF unlocked;
6. Kabaw unlocked;

4.1 The block motions as modeled in six experiments

Table S5. Interseismic velocities of blocks and RMS of observed and modeled velocities

**Block**	V_E, (North) mm/y	V_N, (East) mm/y	ω*, angular velocity, 10^{-6} rad/y, clockwise negative
**Experiment 1 All faults locked**			**RMS observed and modeled velocities 2.5 mm/yr**
Block 1	-2.64	-5.44	-0.012
Block 2	-8.38	-7.93	-0.010
Block 3	-11.65	-15.97	-0.004
**Experiment 2 IBD unlocked**			**RMS observed and modeled velocities 7.1 mm/yr**
Block 1	-10.32	-8.25	-0.009
Block 2	-13.14	-10.95	-0.0099
Block 3	-13.88	-17.46	-0.0047
**Experiment 3 Tripura section of IBD unlocked**			**RMS observed and modeled velocities6.5 mm/yr**
Block 1	-8.82	-7.12	-0.0016
Block 2	-12.24	-10.53	-0.0055
Block 3	-13.01	-17.03	-0.0008
**Experiment 4 Kaladan unlocked**			**RMS observed and modeled velocities 5.1 mm/yr**
Block 1	-0.75	-3.89	-0.0058
Block 2	-13.34	-11.45	-0.011
Block 3	-14.1	-17.69	-0.006
**Experiment 5 CMF unlocked**			**RMS observed and modeled velocities 3.6 mm/yr**
Block 1	-1.78	-4.91	-0.011
Block 2	-8.23	-8.17	-0.008
Block 3	-13.71	-17.82	-0.0012
**Experiment 6 Kabaw unlocked**			**RMS observed and modeled velocities 3.6 mm/yr**
Block 1	-1.53	-5.01	-0.091
Block 2	-7.34	-9.29	-0.033
Block 3	-7.91	-14.68	-0.0017

* Rotation around the geometrical center of block bottoms. Velocities that differ significantly (>3mm/y) from observation are highlighted in pink.

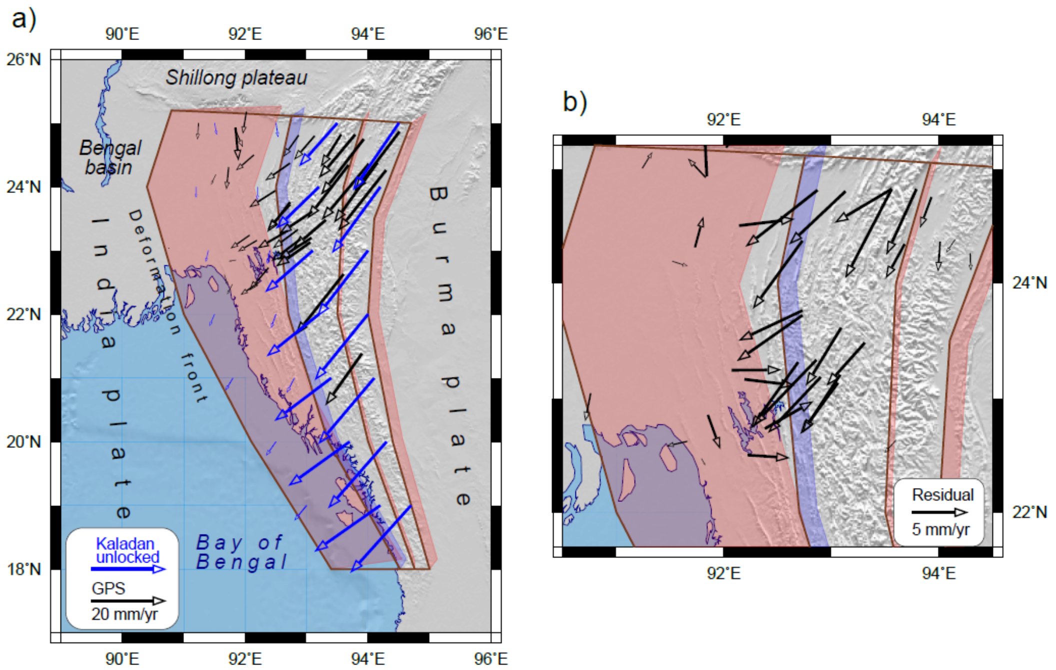

Fig. S5. Block velocities modeled in Experiment 4 "Kaladan fault is unlocked" (blue arrows in (**a**)); GPS velocities are shown by black arrows; locked and unlocked faults are highlighted by transparent red and blue. (**b**) The residual velocities, RMS=5.1mm/y

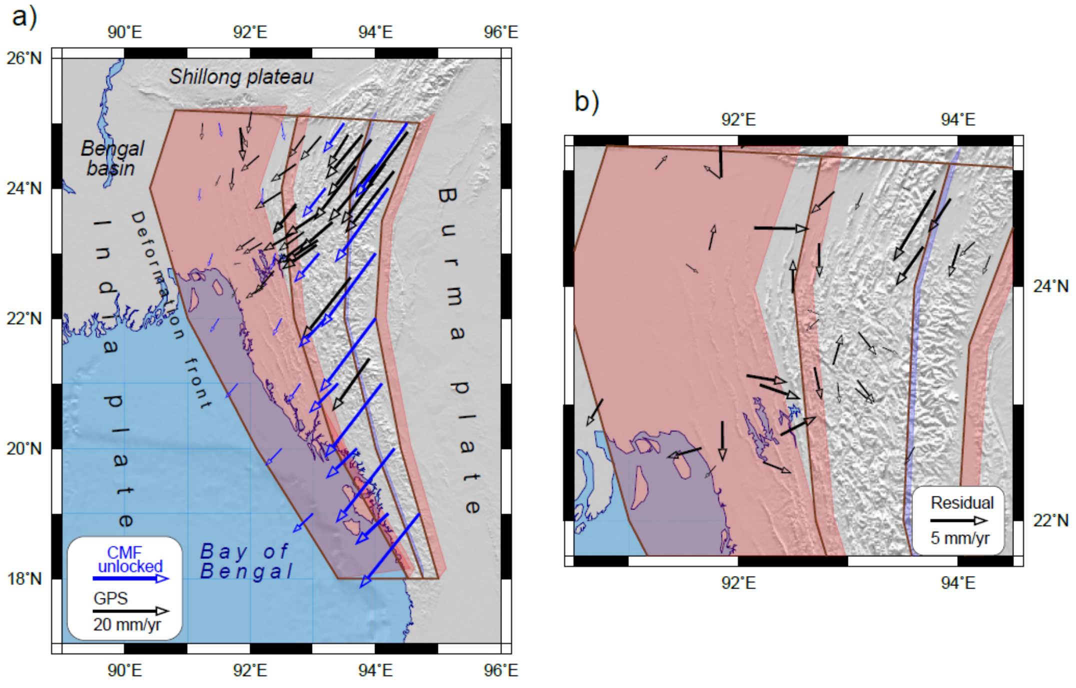

Fig. S6. Block velocities modeled in Experiment 5 "CMF fault is unlocked" (blue arrows in (**a**)); GPS velocities are shown by black arrows; locked and unlocked faults are highlighted by transparent red and blue. (**b**) The residual velocities, RMS=3.1 mm/y

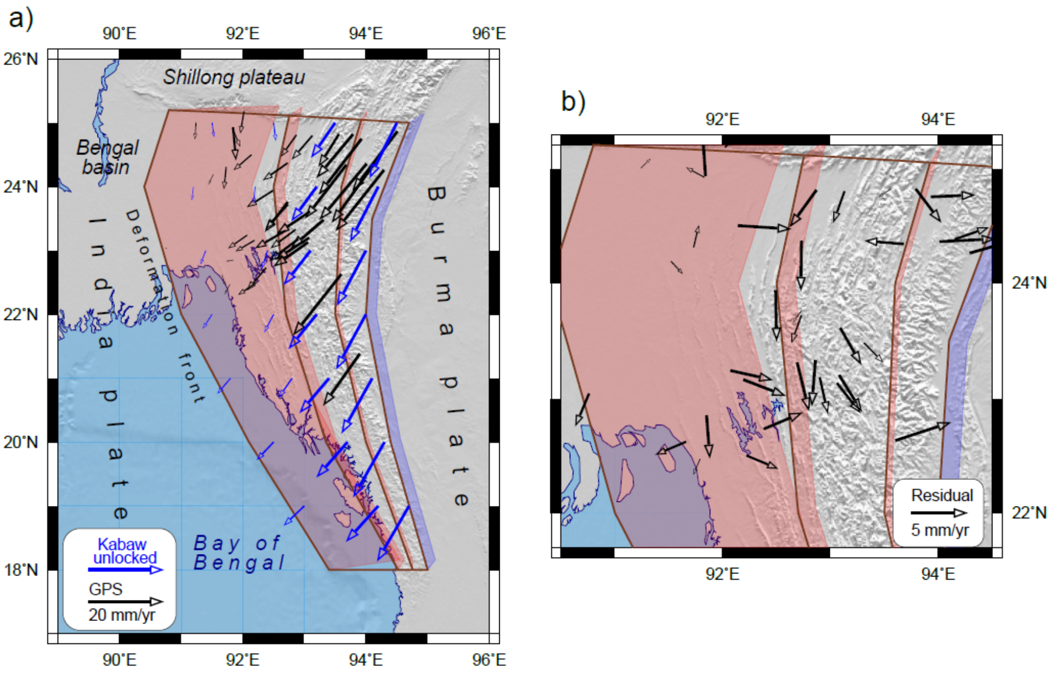

Fig. S7. Block velocities modeled in Experiment 6 “Kabaw fault is unlocked” (blue arrows in (**a**)); GPS velocities are shown by black arrows; locked and unlocked faults are highlighted by transparent red and blue. (**b**) The residual velocities, RMS=3.6mm/y

Velocity maps for experiments 1, 2 and 3 are in the main text, figure 2.

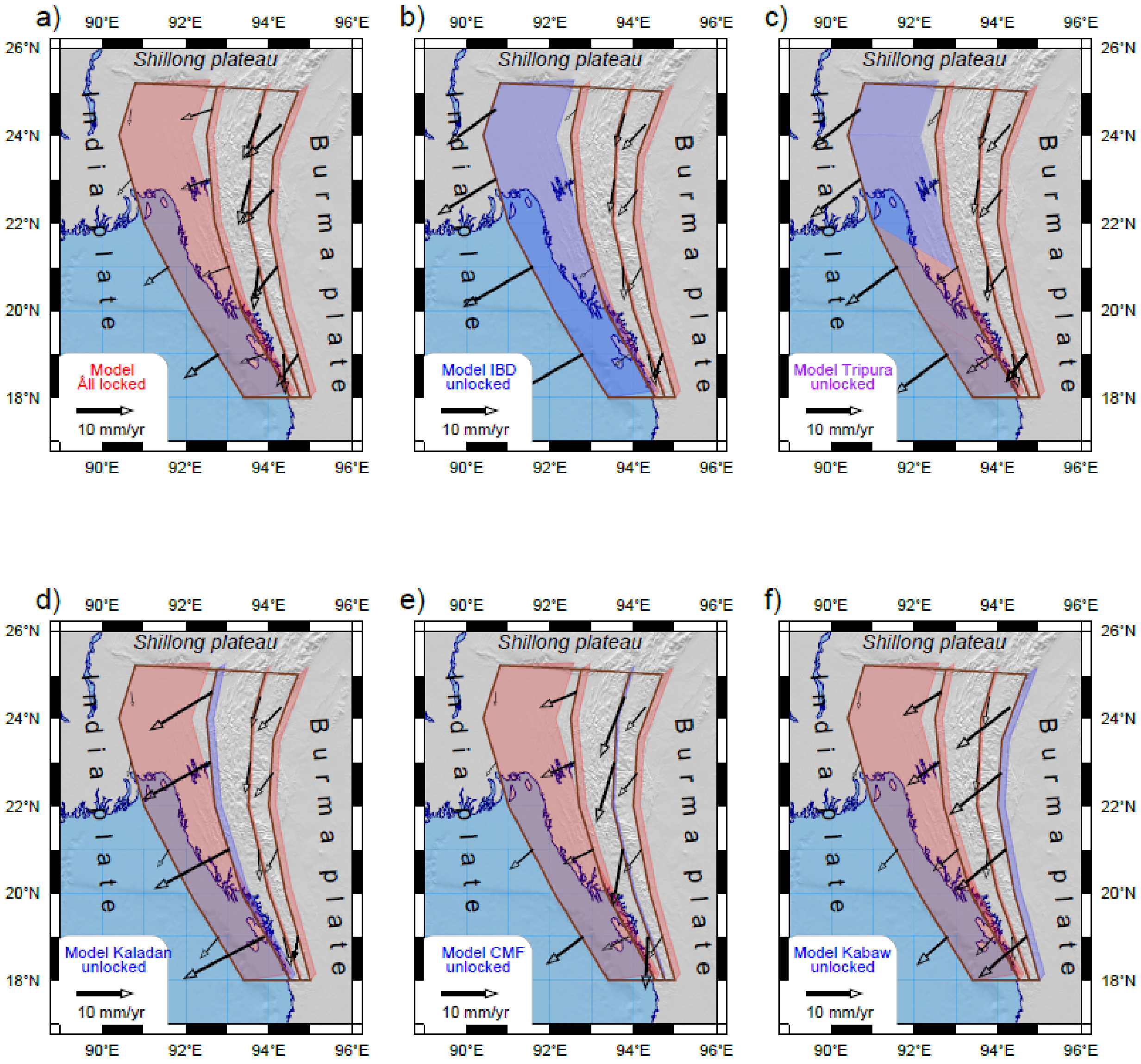

Fig. S8. Relative velocities of right block relatively to the left block in the faults modeled in six Experiments. Locked and unlocked faults are highlighted by transparent red and blue.

4.2 Coupling in the preferred experiment.

The fault coupling is the portion of relative motion released by earthquakes. The displacement at a point (*x*, *y*) of a fault plane is:

$$D_f(x,y) = \sqrt{D_t^2(x,y) + \left(\frac{D_l(x,y)}{\cos\alpha}\right)^2} \qquad \text{(S9)}$$

where the vector (D_t, D_l) is relative displacement of the blocks separated by the given fault; D_t, D_l are along the fault line in the surface, and normal to fault line components, and α is dip angle of the fault. Using total displacement of blocks including rotation, we calculated the displacement D_f

in each cell. Then we calculated total moment of relative displacement in the given segment M_s, and moment of earthquakes M_e:

$$M_s = \sum_{cell} SD_f \; ; \quad M_e = \sum_{earthquakes} AD_e \qquad (s10)$$

where S is cell area, and the sum is taken over all cells of the segment; A is rupture area, D_e.is coseismic displacement, and sum is taken over all earthquakes in the segment The coupling is M_e/M_s, the ratio of the moment released by earthquakes to the total moment the given segment. We calculate earthquake moment using rupture area and coseismic displacement.

Table S6. Coupling in four faults of the Indo-Burman arc in the preferred experiment "All faults locked"

**Fault**	**IBD**		**Kaladan**		**CMF**		**Kabaw**	
	s1	0.60	s5	0.91	s9	0.81	s13	0.83
	s2	0.63	s6	0.94	s10	0.32	s14	0.82
	s3	0.76	s7	0.97	s11	0.06	s15	0.79
	s4	0.91	s8	0.89	s12	0.44	s16	0.87

We obtain high coupling in the IBD, Kaladan and Kabaw, while the coupling in the central segment of CMF is low.

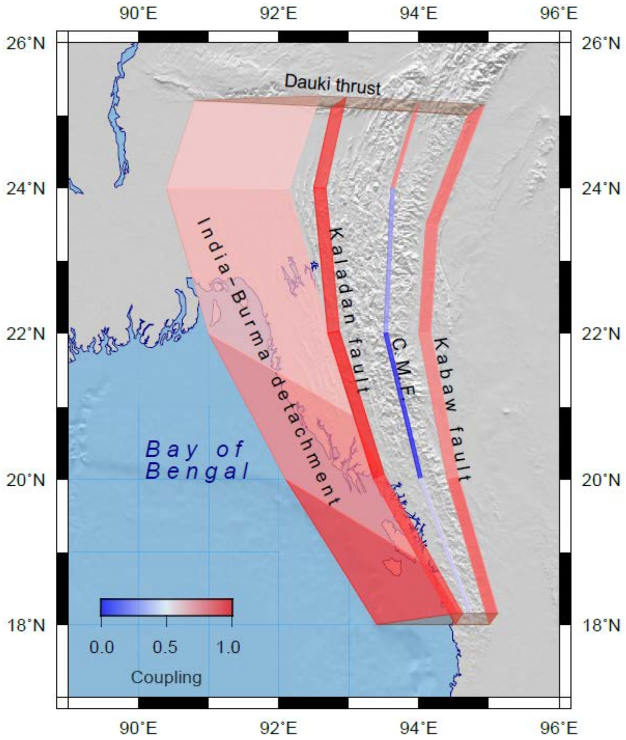

Fig. S9. Coupling in the four faults obtained in the preferred experiment "All faults locked"

4.3 Temporal sequences of earthquakes in the IBD.

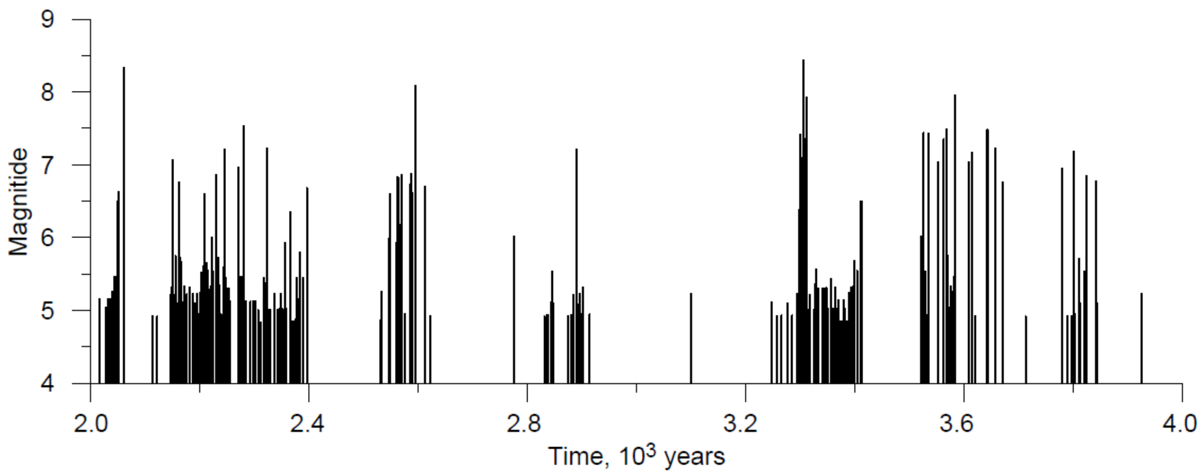

Fig. S10. An irregular pattern of earthquake occurrence in the IBD. Two thousand year long time sequence of all earthquakes generated in preferred Experiment 1 “All locked”.

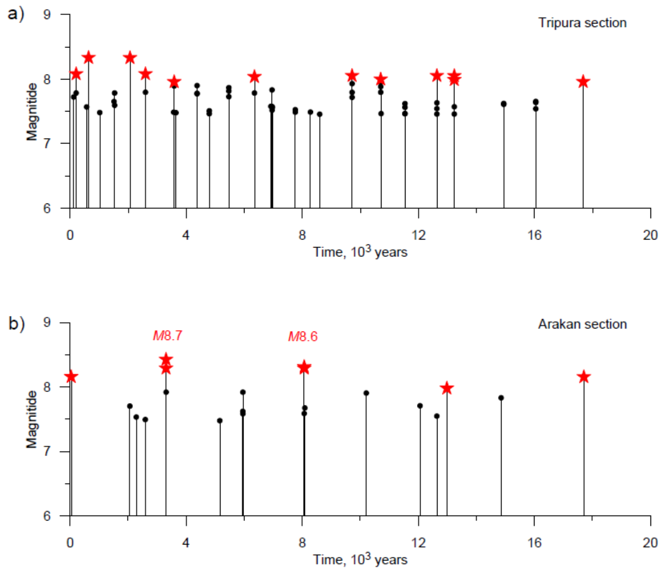

Fig. S11. Time sequence of large (M7.5+) earthquakes in the onshore Tripura section of IBD (a), and in the oceanic Arakan section of IBD (b) in the preferred experiment “All faults locked”. Red stars are M≥8.0

4.4Synthetic seismicity modeled in experiments 2-6.

Table S7. Maximum magnitudes modeled for individual faults in six experiments.

**Experiment**		**All locked**	**IBD unlocked**	**Tripura unlocked**	**Kaladan unlocked**	**CMF unlocked**	**Kabaw unlocked**
**Fault, segment**		**Maximum magnitude**					
**IBD**	**s1**	8.3	-	-	7.6	8.4(8.7*)	8.2
	**s2**	8.1	-	-	7.1	8.4	8.2
	**s3**	8.4(8.7*)	-	7.7	6.1	8.2(8.6*)	7.6
	**s4**	8.3	-	7.9	-	8.3	8.1
**Kaladan**	**s5**	7.7	5.4	7.7	-	7.1	6.7
	**s6**	7.4	6.3	7.7	-	6.9	6.6
	**s7**	7.2	-	7.7	-	6.5	6.4
	**s8**	7.6	-	7.9	-	7.0	6.7
**CMF**	**s9**	7.5	7.4	7.5	7.3	-	7.5
	**s10**	6.1	7.3	7.2	7.3	-	7.1
	**s11**	7.1	6.6	6.1	6.6	-	6.5
	**s12**	7.4	7.6	7.5	7.6	-	7.5
**Kabaw**	**s13**	7.5	7.6	7.4	7.5	6.7	-
	**s14**	7.8	7.8	7.7	7.8	6.8	-
	**s15**	7.7	7.9	7.6	7.9	6.6	-
	**s16**	7.9	7.9	7.9	7.9	6.8	-

*Magnitude of multi-segment earthquake.

Maximum magnitude obtained in each experiment is highlighted in red

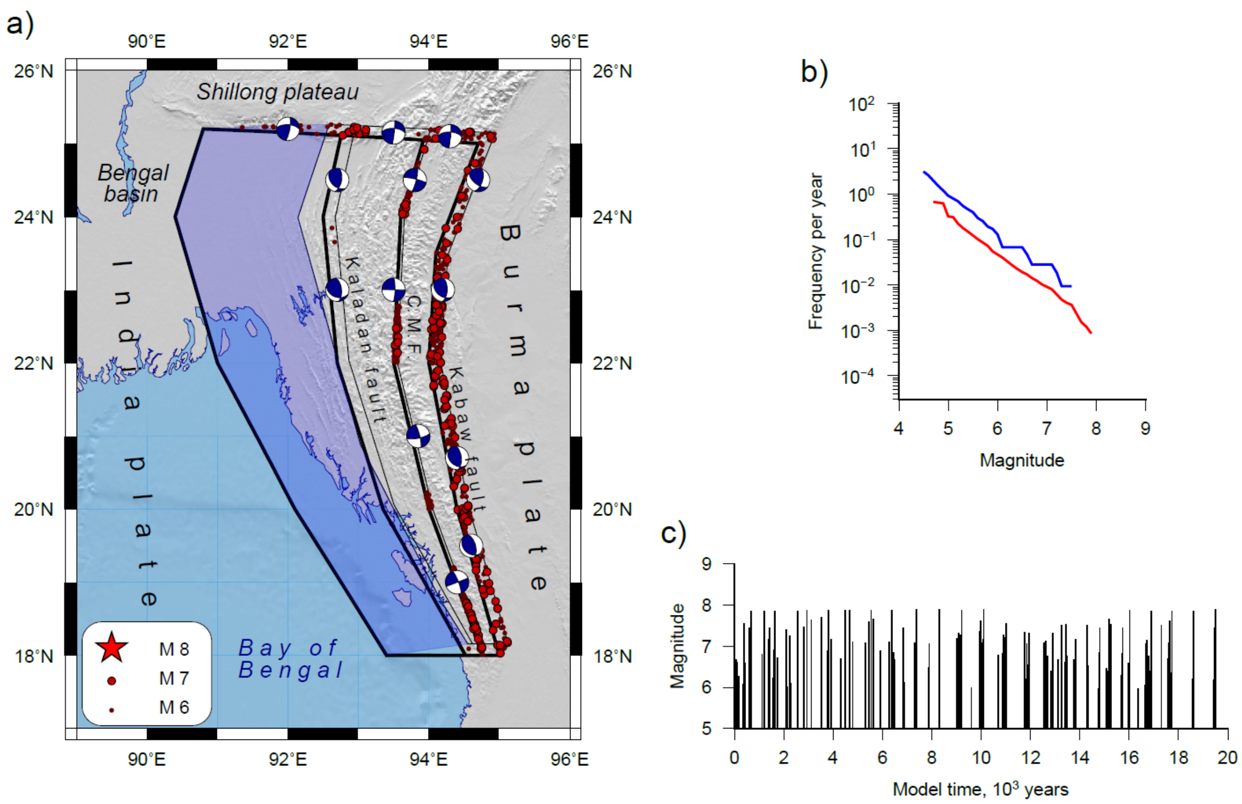

Fig. S12. Overview of synthetic seismicity simulated for 20 thousand years in experiment 2 "IBD unlocked". (**a**) The map of epicenters: unlocked fault is highlighted by transparent blue; beach balls represent synthetic FPS. (**b**) Earthquake size distribution (frequency per year) of the synthetic (red) and recorded (blue) seismicity. (**c**) Time sequence of synthetic earthquakes with M≥6.

No great earthquake with magnitude M≥8 has been produced in this simulation, with the maximum magnitude being 7.9 in the Kabaw fault. The level of seismic activity decreased by a factor of 3-5, while the slope of the earthquake size distribution did not change. The Kaladan fault is almost aseismic, no synthetic earthquakes are generated in the southern segments s7, s8, and a few in the northern segments s5, s6 (Table S7).The seismicity in CMF and Kabaw does not change. Reverse faulting is in the Kaladan and southern segments of Kabaw, while the dextral strike-slip is in CMF and the northern Kabaw segment.

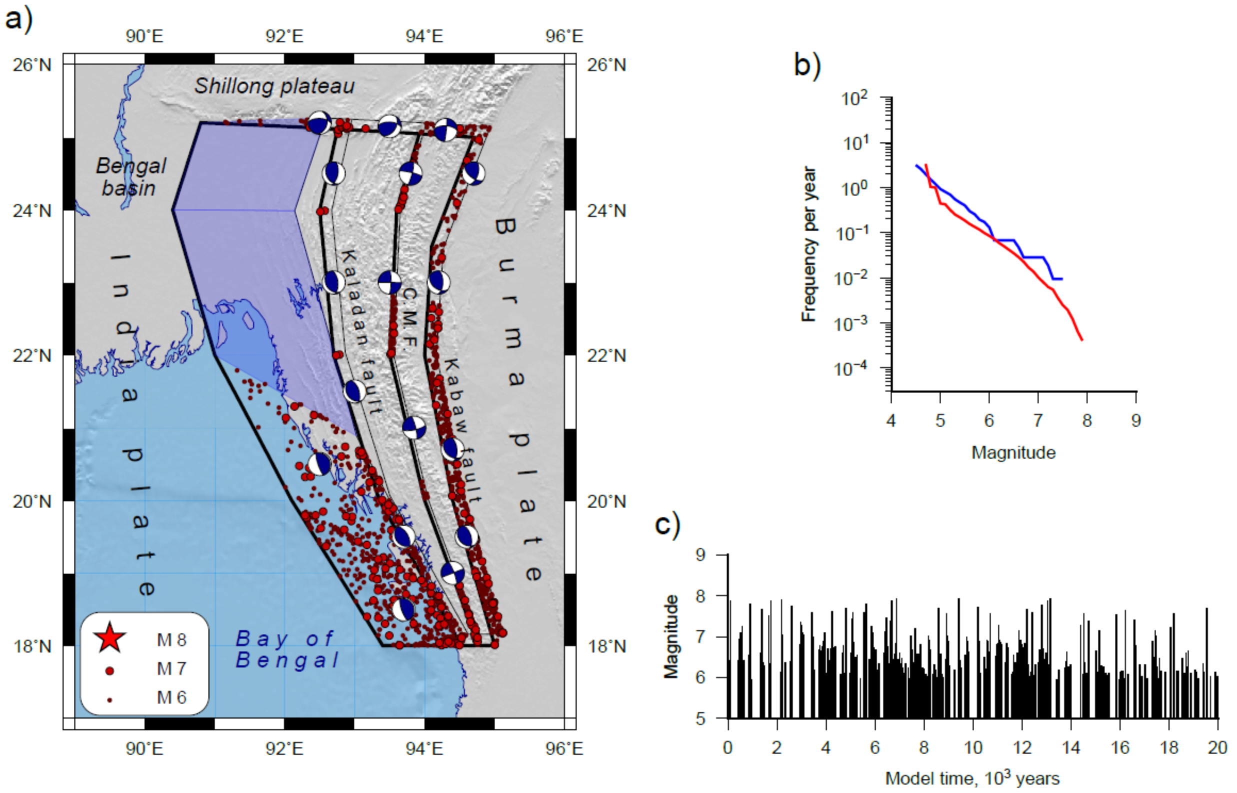

Fig.S13. Overview of synthetic seismicity simulated for 20 thousand years in experiment 3 “Tripura unlocked”. (**a**) The map of epicenters: the unlocked fault is highlighted by transparent blue; beach balls represent synthetic FPS. (**b**) Earthquake size distribution (frequency per year) of the synthetic (red) and recorded (blue) seismicity (**c**) Time sequence of synthetic earthquakes with M≥6.

No great earthquake with magnitude M≥8 has been produced in this simulation, the maximum magnitude is 7.9 in the south of IBD, Kabaw, and Kaladan faults. The level of seismic activity decreased by a factor of 2-3, while the slope of the earthquake size distribution did not change. The north of the Kaladan fault is almost aseismic (Table S7). The seismicity in CMF and Kabaw did not change. Reverse faulting is in the IBD, Kaladan and southern segments of Kabaw, while the dextral strike-slip is in CMF and northern segment of Kabaw.

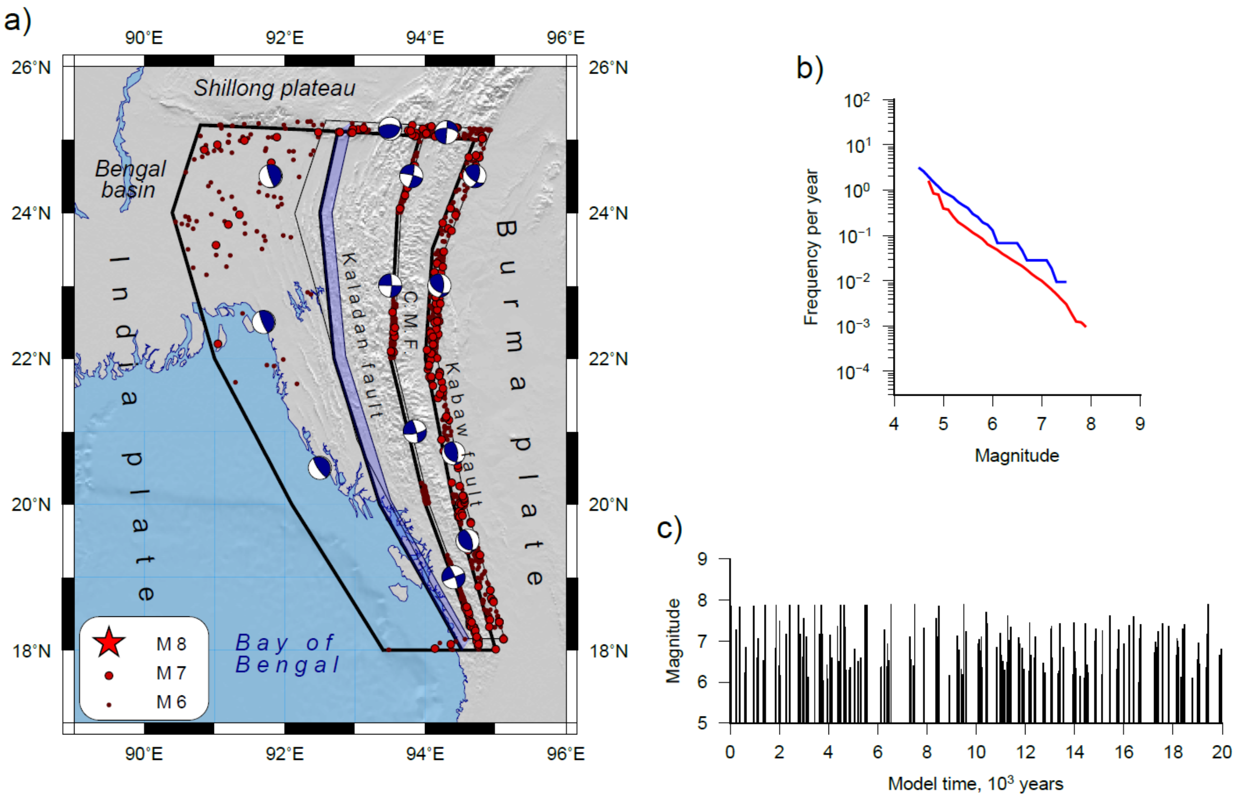

Fig.S14. Overview of synthetic seismicity simulated for 20 thousand years in experiment 4 “Kaladan unlocked”. (**a**) The map of epicenters: the unlocked fault is highlighted by transparent blue; beach balls represent synthetic FPS. (**b**) Earthquake size distribution (frequency per year) of the synthetic (red) and recorded (blue) seismicity (**c**) Time sequence of synthetic earthquakes with M≥6.

No great earthquake with magnitude M≥8 has been produced in this simulation. The maximum magnitude is 7.9 in the Kabaw fault. The level of seismic activity decreased by a factor of 3, while the slope of the earthquake size distribution did not change. The southern segments s3, s4 of IBD are aseismic, and few earthquakes were simulated in the northern segments s1, s2 (Table S7). Seismicity in CMF and Kabaw did not change. Reverse faulting is in the IBD and south of Kabaw, dextral strike-slip is in CMF and northern segment of Kabaw

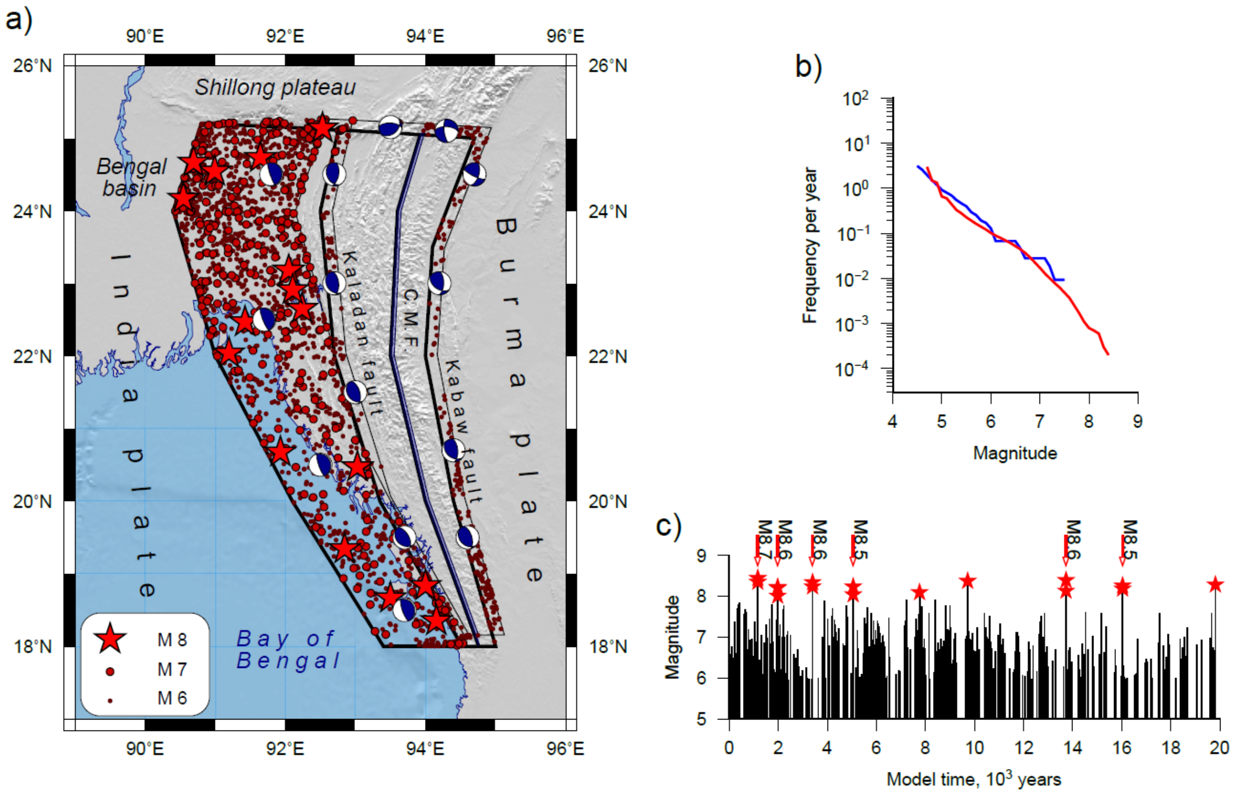

Fig.S15. Overview of synthetic seismicity simulated for 20 thousand years in experiment 5 "CMF unlocked". (**a**) The map of epicenters: the unlocked fault is highlighted by transparent blue. Beach balls represent synthetic FPS (**b**) Earthquake size distribution (frequency per year) of the synthetic (red) and recorded (blue) seismicity (**c**) Time sequence of synthetic earthquakes with M≥6.

The rate of seismicity decreased insignificantly compared with the preferred experiment, and the slope of the earthquake size distribution did not change. The IBD seismicity is similar to the one obtained in the preferred experiment. Sixteen great earthquakes with magnitude M≥8 have been produced in this simulation in the IBD with maximum M=8.4. Six multi-segment earthquakes had magnitude M≥8.5; four of them occurred in Tripura (M_{max}=8.7), and two in Arakan (M_{max}=8.6). The seismic activity and maximum magnitude decreased in the Kabaw and Kaladan faults compared with the preferred experiment (Table S7). Reverse faulting is in the IBD, Kaladan and south of Kabaw, while the dextral strike-slip is in the northern segment of Kabaw.

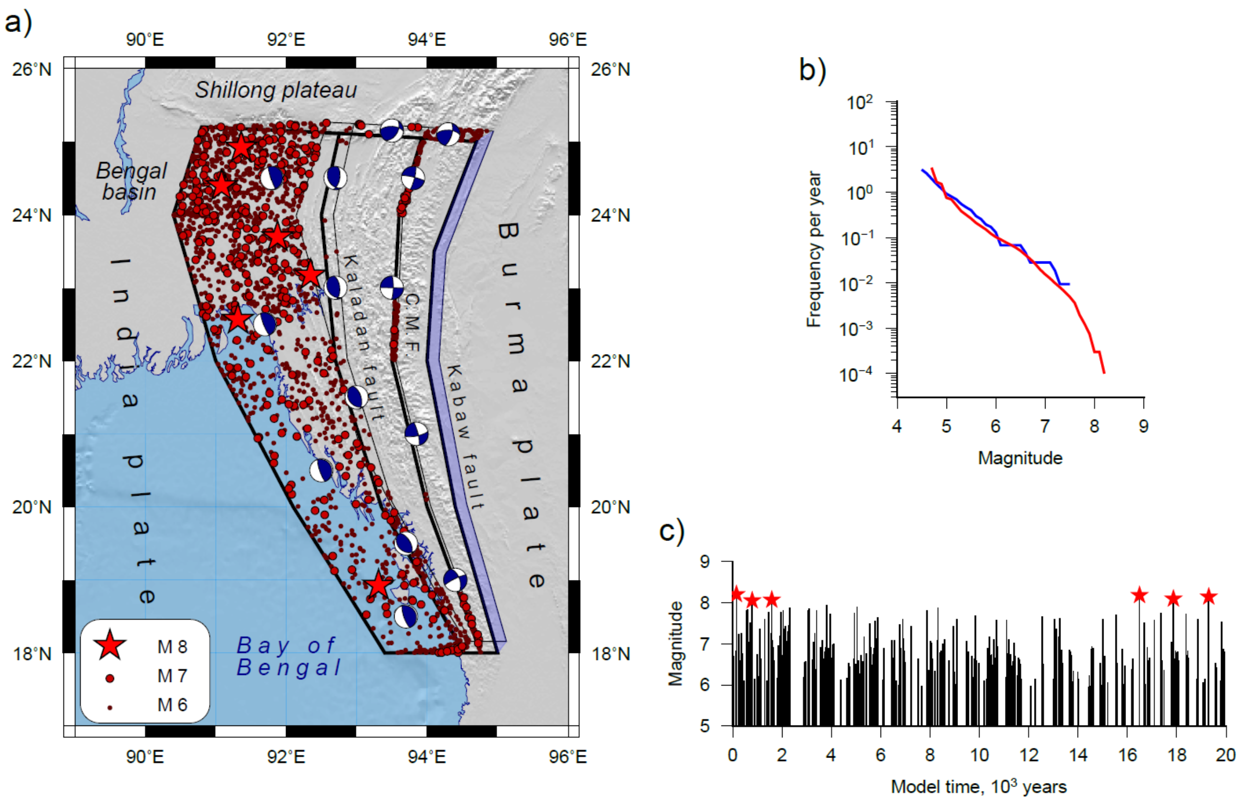

Fig.S16. Overview of synthetic seismicity simulated for 20 thousand years in experiment 6 "Kabaw unlocked". (**a**) The map of epicenters: the unlocked fault is highlighted by transparent blue. Beach balls represent synthetic FPS. (**b**) Earthquake size distribution (frequency per year) of the synthetic (red) and recorded (blue) seismicity (**c**) Time sequence of synthetic earthquakes with M≥6.

Six great earthquake with magnitude M≥8 have been produced in this simulation in the IBD; the maximum magnitude 8.2 was in Tripura. The level of seismic activity and the slope of the earthquake size distribution are comparable with those obtained in the preferred experiment. The number of earthquakes and the maximum magnitude in Kaladan decreased significantly (Table S7). Reverse faulting is in the IBD and Kaladan, while the dextral strike-slip is in CMF

5.Experiment 7 “Including the Sagaing fault”.

We performed an additional numerical experiment for the block structure including the Sagaing fault and supposing all the faults to be locked, like in the preferred experiment. The Central Myanmar basin is being transformed from an outer block to an inner one. We set the basal velocity (-17; -22) under the Myanmar basin, and external velocity of the Shan plateau (-18; -42) using the observed GPS measurements (Steckler et al., 2016, Vernant et al 2014)).

5.1 Block motions

The comparison of the interseismic velocities obtained after including of the Sagaing fault with the preferred experiment shows that velocities have changed insignificantly. (Figure S17 and Table S8). The RMS of the modeled and observed velocities is 2.8mm/y.

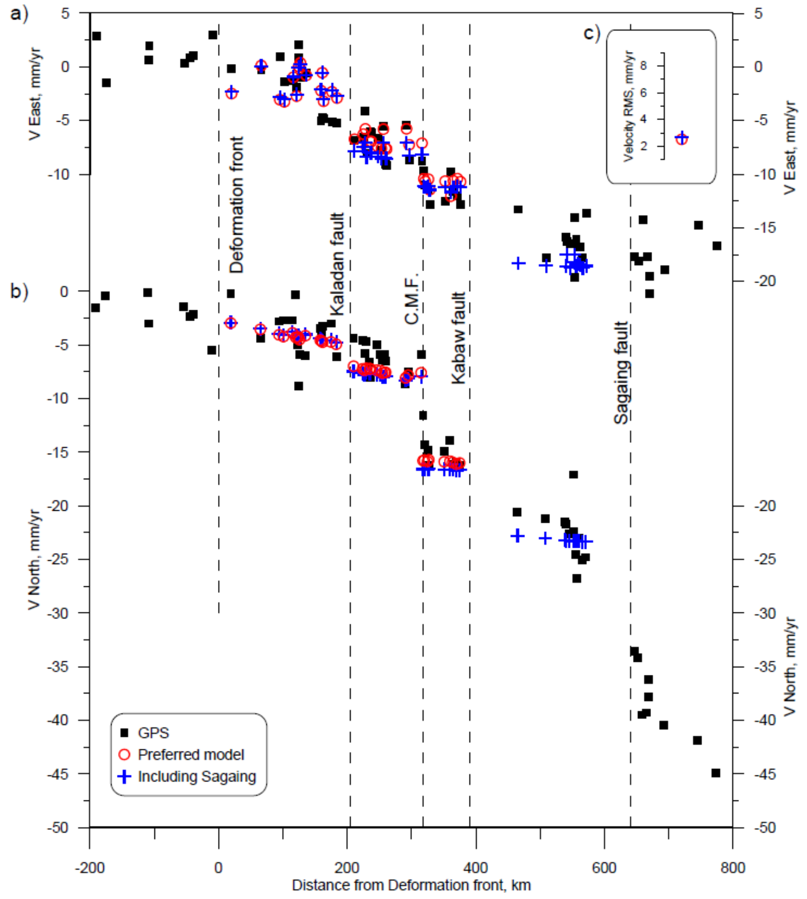

Fig. S17. Changes of the modeled interseismic velocities across the Indo-Burman arc simulated in the preferred Experiment 1 in the Experiment 7 “Including the Sagaing Fault”. The values are given at the GPS sites, their locations in the plot are distances to the nearest fault. Fault locations (dashed lines) are as at 24°N. The modeled velocities are shown by colored symbols; observed GPS velocities (Table S1) are shown by black squares. (**a**), Eastward velocities; (**b**), Northward velocities; (**c**), Residual Mean Squared velocity, symbols are the same as in (**a**) and (**b**).

Table S8. Interseismic velocities of blocks in the preferred model (experiment 1), and model including Sagaing (Experiment 7).

Block	*V*x, (North) mm/yr	*V*y, (East) mm/yr	*ω*, angular velocity, 10^{-6} rad/yr clockwise negative
**Experiment 1**	**All faults locked**		**RMS 2.5mm/y**
**Block 1**	-2.64	-5.44	-0.012
**Block 2**	-8.38	-7.93	-0.010
**Block 3**	-11.65	-15.97	-0.004
**External Block Myanmar basin**	-17.00	-22.00	0
**Experiment 7**	**Including Sagaing**		**RMS 2.8mm/y**
**Block 1**	-2.47	-5.21	-0.011
**Block 2**	-9.15	-8.22	-0.008
**Block 3**	-11.54	-16.65	-0.0014
**Block 4 Myanmar basin**	-17.83	-22.98	-0.005

5.2 Synthetic seismicity

The model generates about 125,000 synthetic earthquakes M≥4.7, among them 50,000 are in the Sagaing fault. The maximum magnitude in the Sagaing is 7.8 (Table S9), and 60 events have magnitude M≥7.5. No multi-segment earthquakes are modeled in the Sagaing.

Comparison of the synthetic seismicity in the IBD, the Kaladan, the CMF, and the Kabaw faults with the seismicity obtained in the preferred experiment does not show significant difference. We note some growth of seismicity in the IBD and some decreasing in the other faults (Table S9). All 25 great synthetic earthquakes (*M*8+) occurred in the IBD: 12 events in the Tripura and 13 events in the Arakan. The maximum magnitude 8.6 is modeled in the Tripura. We obtain 6 multi-segment mega-earthquakes: two in the Arakan section (*M*8.6 and 8.5), three in the Tripura section (*M*8.8, 8.6 and 8.5), and one mega-earthquake with magnitude *M*=8.9 ruptured most of IBD excluding most southern segment (Figure S18a).

The recurrence time of M8+ earthquakes is shown in the figure S19. Compared to the preferred experiment (Figure S11), the recurrence interval has shortened in the Arakan section, This is probably due to a drop of seismic activity in the Kaladan and Kabaw faults. In Tripura the recurrence time has not changed but maximum magnitudes have increased. The recurrence time of great earthquakes is irregular.

In accordance with our expectation, the transformation of the Myanmar basin from an outer block to an inner one has little effect on the seismicity in the IBD. It is not surprising, because the motion of the Central Myanmar block is almost the same, whatever is it the outer or the inner block, and consequently motions of all other block have little change**.** In addition, we reproduce high seismic activity in the Sagaing fault that validate performance of the BAFD in the modeling of the complex regions with various style of faulting.

Table S9. Comparison maximum magnitudes in the individual faults in the preferred model (Experiment 1), and in the model including Sagaing (Experiment 7).

**Experiment**		**All locked preferred model**	**All locked including Sagaing**
**Fault, segment**			
**IBD**	**s1**	8.3	8.4
	**s2**	8.1	8.6(8.9*)
	**s3**	8.4(8.7*)	8.3(8.6*)
	**s4**	8.3	8.3
**Kaladan**	**s5**	7.7	7.1
	**s6**	7.4	6.8
	**s7**	7.2	7.0
	**s8**	7.6	7.5
**CMF**	**s9**	7.5	7.3
	**s10**	6.1	6.6
	**s11**	7.1	-
	**s12**	7.4	7.1
**Kabaw**	**s13**	7.5	7.7
	**s14**	7.8	7.5
	**s15**	7.7	7.2
	**s16**	7.9	7.0
**Sagaing**	**s17****	-	7.7
	**s18****	-	7.7
	**s19****	-	7.8
	**s20****	-	7.8

*Magnitude of multi-segment earthquake.
**Segments of the Sagaing Fault are numbered from north to south.
Maximum magnitude obtained in each experiment is highlighted in red

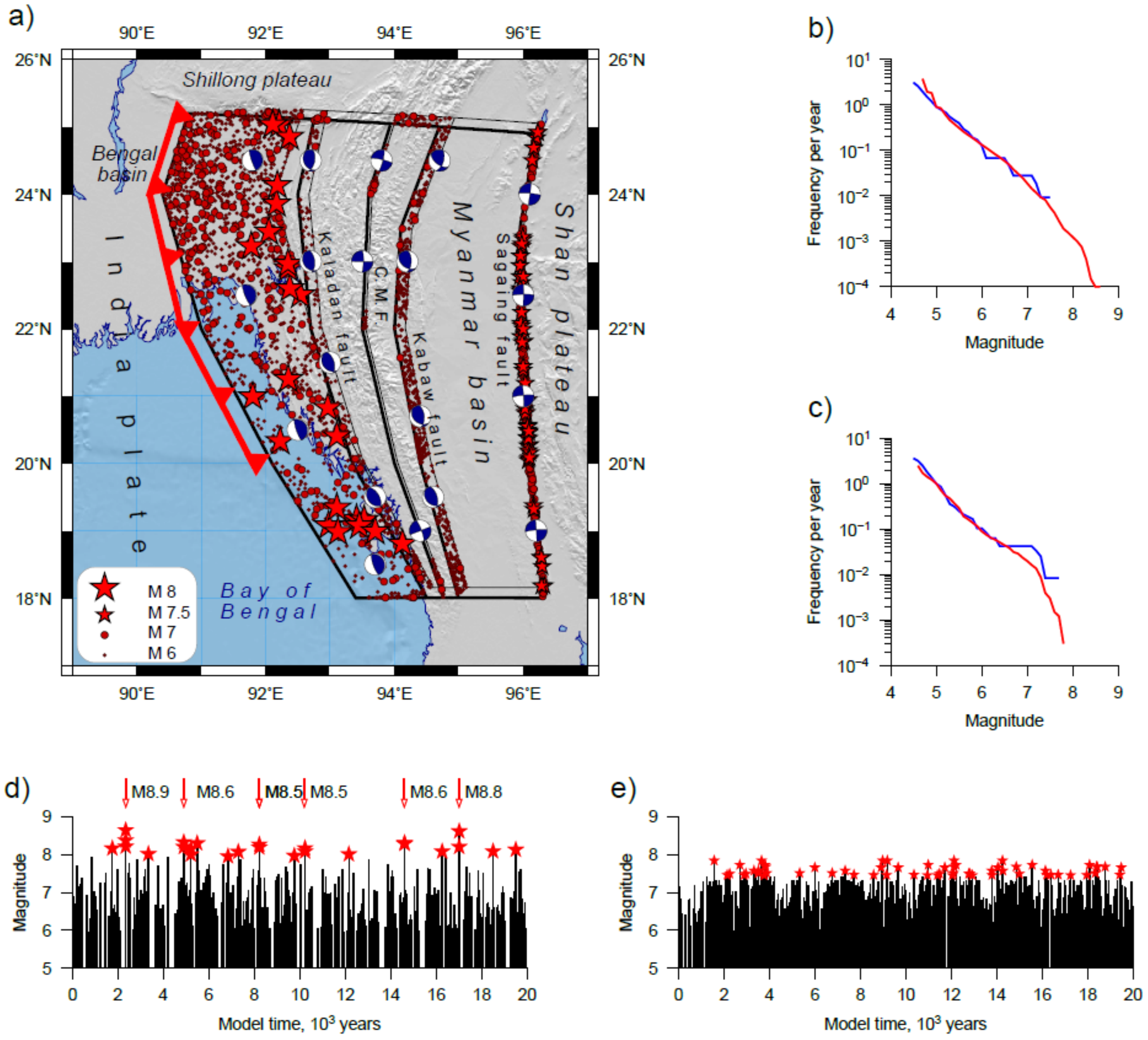

Fig. S18. Overview of synthetic seismicity simulated for 20 thousand years in the experiment 7 "Including of the Sagaing". (**a**) The map of epicenters: stars are the largest earthquakes M8+ in the IBD and M7.5+ in the Sagaing; beach balls represent synthetic FPS. (**b, c**) Earthquake size distributions (frequency per year) of the synthetic (red) and recorded (blue) seismicity in the faults included in the preferred experiment (**b**) and in the Sagaing (**c**). (**d, e**) Time sequences of synthetic earthquakes with M≥6 in the faults included in the preferred experiment (**d**), and in the Sagaing (**e**). Arrows in (**d**) point to multi-segment great earthquakes. The red thrust line shows the rupture zone of maximum modeled mega-earthquake with M=8.9.

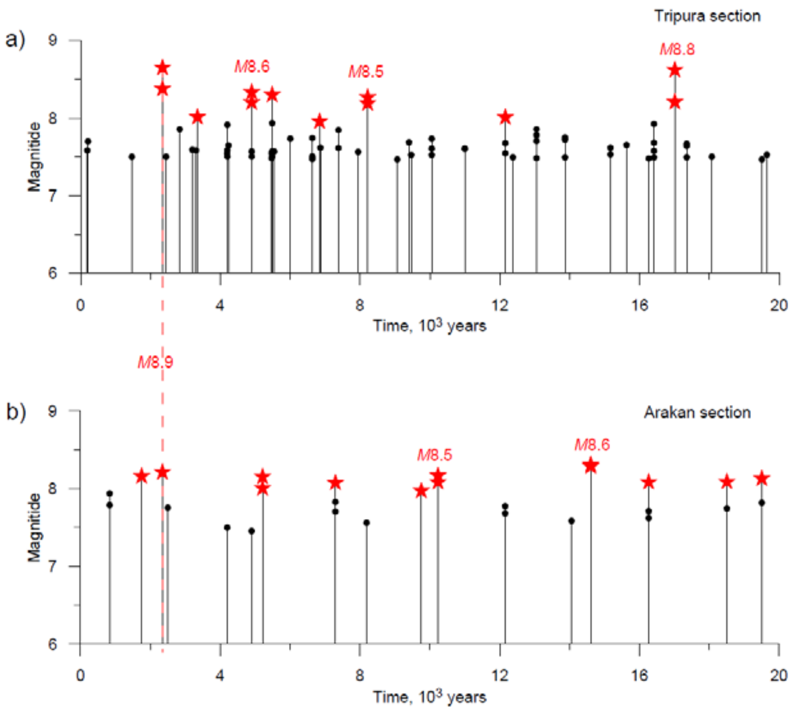

Fig. S19. Time sequence of large (*M*7.5+) earthquakes in the onshore Tripura section of IBD **(a)**, and in the oceanic Arakan section of IBD **(b)** in the experiment “Including Sagaing”. Red stars are *M*≥8.0 and multi-segment earthquakes are labeled. Red dashed line is mega-earthquake with magnitude *M*8.9

References

1. Steckler, M. S., Mondal, D. R., Akhter, S. H., Seeber, L., Feng, L., Gale, J., et al. (2016). Locked and loading megathrust linked to active subduction beneath the Indo -Burma Ranges. Nature Geoscience, 9(8), 615–618. https://doi.org/10.1038/ngeo2760. https://doi.org/ 10.1038/ngeo2760.
2. Gahalaut, V. K., Kundu, B., Laishram, S. S., Catherine, J., Kumar, A., Singh, M. D., et al. (2013). Aseismic plate boundary in the Indo- Burmese wedge, northwest Sunda Arc. Geology, 41(2), 235–238. https://doi.org/10.1130/G33771.1
3. Mallick, R., Lindsey, E. O., Feng, L., Hubbard, J., Banerjee, P., & Hill, E. M. (2019). Active convergence of the India- Burma-Sunda plates revealed by a new continuous GPS network. Journal of Geophysical Research: Solid Earth, 124, 3155–3171. https://doi.org/10.1029/ 2018JB016480
4. Vernant, P., R. Bilham, W. Szeliga, D. Drupka, S. Kalita, A. K. Bhattacharyya, V. K. Gaur, P. Pelgay, R. Cattin, and T. Berthet (2014), Clockwise rotation of the Brahmaputra Valley relative to India: Tectonic convergence in the eastern Himalaya, Naga Hills, and Shillong Plateau, J. Geophys. Res. Solid Earth, 119, doi:10.1002/2014JB011196.
5. Maurin, T., Masson, F., Rangin, C., Min, U. T., & Collard, P. (2010). First global positioning system results in northern Myanmar: Constant and localized slip rate along the Sagaing fault. Geology, 38(7), 591–594. https://doi.org/10.1130/G30872.1
6. Banerjee, P., R. Bürgmann, B. Nagarajan, and E. Apel (2008), Intraplate deformation of the Indian subcontinent, Geophys. Res. Lett., 35, L18301, doi:10.1029/2008GL035468.
